# Genetic Trends in General Combining Ability for Maize Yield-Related Traits in Northeast China

**DOI:** 10.3390/cimb47110877

**Published:** 2025-10-23

**Authors:** Haochen Wang, Xiaocong Zhang, Jianfeng Weng, Mingshun Li, Zhuanfang Hao, Degui Zhang, Hongjun Yong, Jienan Han, Zhiqiang Zhou, Xinhai Li

**Affiliations:** 1 College of Agriculture, Heilongjiang Bayi Agricultural University, Daqing 163000, China; whcnky@163.com; 2 State Key Laboratory of Crop Gene Resources and Breeding, Institute of Crop Sciences, Chinese Academy of Agricultural Sciences, Beijing 100081, China; cong_11101@126.com (X.Z.); wengjianfeng@caas.cn (J.W.); limingshun@caas.cn (M.L.); haozhuanfang@163.com (Z.H.); zhangdegui@caas.cn (D.Z.); yonghongjun@caas.cn (H.Y.); hanjienan@caas.cn (J.H.); 3 Heilongjiang Academy of Agricultural Sciences, Harbin 150086, China

**Keywords:** general combining ability (GCA), breeding process, parents and offspring, yield traits

## Abstract

Maize (*Zea mays* L.) is the most extensively cultivated food crop in China, and current studies on maize general combining ability (GCA) focus primarily on the genetic basis of traits. However, the dynamic trends and underlying genetic loci associated with GCA for yield-related traits during breeding remain underexplored. This study was designed to investigate the changing trends of the general combining ability (GCA) and the frequency of elite alleles among 218 major maize inbred lines from Northeast China, spanning the 1970s to the 2010s. PH6WC and PH4CV were used as testers to develop 436 hybrid combinations via the North Carolina design II (NCII) method, and these combinations were evaluated across three environments. We further analyzed the combining ability (particularly the GCA) of 16 yield-related traits and their dynamic trends during breeding, grouped into three age periods (AGE1: 1960s–1970s; AGE2: 1980s–1990s; AGE3: 2000s–2010s). We also screened for genetic loci associated with the GCA effects of these traits. Results show that breeding selection significantly affected the GCA of six yield-related traits (ear length (EL), tassel branch number (TBN), tassel main axis length (TL), kernel length (KL), stem diameter (SDR), and hundred kernel weight (HKW)). Specifically, the mean TBN_GCA_ value decreased from 2.51 in AGE1 to −1.28 in AGE3, and the mean HKW_GCA_ increased from −1.58 in AGE1 to 0.36 in AGE3. Yield per plant GCA (YPP_GCA_) was positively correlated with the GCA values of EL, ear diameter (ED), kernel row number (KRN), kernel number per row (KNPR), and HKW. Association analysis identified 38 single nucleotide polymorphisms (SNP_S_) related to GCA. The T/T alleles for TBN were absent in AGE1, emerged in AGE2 (1980s–1990s), and persisted in AGE3—consistent with the decreasing trend of TBN_GCA_ from AGE1 to AGE3. For HKW, the A/A alleles not only exhibited higher GCA (effectively improving the HKW_GCA_ of inbred lines) but also showed an 11% increase in allelic frequency from AGE1 to AGE3. Taken together, these results suggest that the accumulation of elite alleles is the primary factor driving the GCA improvement during maize breeding in Northeast China.

## 1. Introduction

Maize is the food crop with the largest planted area in China and serves as the country’s primary cereal crop, functioning as a crucial source of food, feed, and industrial raw materials [1]. According to data from the China Statistical Yearbook, 2024 [2], the national maize planting area in 2024 was 44.74 million hectares, with a total output of 294.92 million tons, accounting for 37.49% of the national total grain sown area and 41.74% of the national total grain output, respectively [3]. In Northeast China, the sowing area of spring maize is approximately 14 million hectares, with a total output of 117.27 million tons, accounting for 31.29% of the national sowing area and 39.76% of national total output of maize, respectively [4]. During the breeding process, this region has accumulated abundant germplasm resources, primarily due to its ecological environment being similar to that of major maize-growing regions in Europe and America, as well as the extensive participation of domestic and international seed companies [5,6,7,8,9]. Spring maize in Northeast China mainly utilizes germplasm resources adapted to cool climates, such as elite genes derived from local landraces through long-term selection, and introduced foreign germplasm with early-maturing and low-temperature-tolerant traits. These germplasm resources perform well in lodging resistance, density tolerance, disease and insect resistance, and other aspects, providing a foundation for the high and stable yield of spring maize. The introduction of diverse germplasm resources is crucial for improving breeding efficiency. Notably, the continuous introduction of favorable alleles—such as those associated with grain moisture content and lodging resistance—has not disrupted the existing heterotic groups in this region [10]. With the continuous advancement of China’s agricultural supply-side structural reform, the most effective approach to maintain the total maize output while adjusting and reducing its planting area is to increase per-unit yield and tap into the crop’s yield potential. Cultivating new maize varieties with high yield, superior quality, and stress resistance represents a critical pathway to enhance per-unit yield [11,12,13,14]. Rich genetic diversity in elite maize germplasm resources serves as the prerequisite and foundation for breeding breakthrough new varieties [15,16,17,18]. However, the narrow genetic base of existing germplasm resources is a major constraint limiting the progress of maize breeding. Therefore, the expansion, improvement, and innovation of maize germplasm resources have become key priorities that require urgent attention in modern maize genetic breeding [18,19].

Genetic improvement of yield traits represents the primary objective of maize breeding. Dissecting the genetic basis underlying the formation of yield traits and their combining ability will provide theoretical support for the development of new maize varieties. Yield traits in crops are quantitative traits controlled by multiple minor genes. The phenotypes of these quantitative traits are influenced by various environmental conditions and genetic components. Compared with single-gene-controlled traits, quantitative traits exhibit a more complex genetic basis [20,21,22,23]. With the continuous advancement of molecular marker-assisted breeding, high-throughput sequencing technologies, and crop genomics, the identification and utilization of genes related to maize yield, quality, and stress resistance have been significantly promoted [23,24]. At present, in the practice of crop genetic breeding, combining ability has been successfully applied to the selection and improvement of maize parental lines, as well as hybrid combinations and other aspects. However, the dynamic trends of combining ability at the breeding stage remain insufficiently explored [25]. A genome-wide association study (GWAS) plays a critical role in linkage marker development, target gene identification, and genetic research on complex traits. It has been widely applied in studies on crop quantitative traits and crop breeding [26,27]. For example, Li et al. [28] performed GWAS on the early flowering trait in maize using a nested association mapping population, identifying 220 candidate genes within a 1 Mb interval around the associated SNPs. These SNPs were concentrated within the candidate genes and within a 5 kb region adjacent to the genes, significantly influencing the early flowering trait in maize. Hu et al. [29] identified 18 candidate genes via GWAS using 282 maize inbred lines, providing a molecular basis for breeding cold-tolerant new varieties. Lv et al. [30] used an NCII design to cross Ye 478 and its near-isogenic line population with three testers, forming 198 hybrid combinations. The results showed that 69 quantitative trait loci (QTL) were detected for yield-related traits, while only 9 QTL were identified for yield per plant GCA (YPP_GC A_), indicating distinct genetic loci between yield-related traits and their GCA. General combining ability (GCA) refers to the average performance of yield (or other quantitative traits) in a series of hybrid combinations, which is derived from a tested inbred line crossed with other inbred lines. Qi et al. [31] adopted an NCII design to develop 300 hybrid combinations. The results revealed 56 QTL for GCA and 21 QTL for specific combining ability (SCA), with only 5 QTL co-controlling both GCA and SCA, confirming distinct genetic bases for GCA and SCA. Additionally, a positive correlation was observed between GCA effect values and significant GCA loci, suggesting that the accumulation of significant GCA loci can effectively enhance trait performance. NCII, first described as the design II mating design or factorial design described by Comstock and Robinson in 1948, is also known as North Carolina design II or AB design. By dividing parents into two groups (usually the “tester group” and “tested line group”), the NCII design only conducts intergroup crossing. This approach greatly reduces the number of hybrid combinations while still enabling efficient and accurate estimation of GCA and SCA, balancing “scientific rigor” and “practicality”. Zhou et al. [32] employed an NCII design to cross two testers with a population of 328 recombinant inbred lines (RILs), conducting QTL analysis for the GCA and SCA of plant height-related traits. The results showed that 21, 30, and 17 QTL were detected in GCA, F_1_ hybrids, and SCA, respectively. Adebayo et al. [33] used 24 drought-tolerant maize inbred lines as experimental materials and adopted an NCII genetic mating design to develop 96 hybrid combinations. The results showed that the GCA effects accounted for 53% of the total phenotypic variation. Despite these advances, current studies on maize combining ability primarily focus on the genetic basis of traits; in contrast, the dynamic trends and underlying genetic loci of combining ability for yield-related traits during breeding remain insufficiently explored.

In this study, the NCII mating design and whole-genome resequencing technology were employed to conduct a GWAS on the combining ability for 218 major maize inbred lines from Northeast China, released by major local breeding institutions from the 1970s to the 2010s. The primary objective was to clarify the trends of combining ability, especially the general combining ability, and identify related loci changes during the breeding process in the Northeast China maize region, thereby providing a theoretical foundation for genetic improvement of high-yield spring maize in Northeast China.

## 2. Materials and Methods

### 2.1. Materials for the Test

A total of 218 major parental inbred lines were selected as experimental materials. These lines are derived from nationally or provincially certified maize single-cross hybrids that have been widely adopted in the spring maize-producing region of Northeast China during the 1970s–2010s. Based on the certification time of the derived hybrids and the breeding time of inbred lines themselves, these 218 inbred lines were categorized into three distinct breeding stages: the early breeding stage (1960s–1970s, designated as AGE1), the mid-breeding stage (1980s–1990s, designated as AGE2), and the recent breeding stage (2000s–2010s, designated as AGE3). Using PH4CV (a core inbred line of the Lancaster heterotic group) and PH6WC (a core inbred line of the Reid heterotic group)—the two dominant heterotic groups in the Northeast China maize region—as tester lines, a total of 436 hybrid combinations were generated following the NCII design (Group A (tester group): 2 tester lines, i.e., PH4CV and PH6WC; Groups B (tested line group): 218 inbred lines; total number of hybrid combinations= Group A size × Group B size = 2 × 218 = 436).

### 2.2. Experimental Methods

The NCII genetic mating design was implemented, with two tester lines (PH4CV and PH6WC) utilized. Notably, these two lines are the parental inbred lines of commercially successful maize hybrid Xianyu 335, with PH6WC designated as the female parent and PH4CV as the male parent. A total of 218 inbred lines were used as tested lines, and 436 hybrid combinations were generated through controlled pollination during 2016–2017. In 2018, these combinations were subjected to field trials at three ecologically distinct locations in Northeast China: (1) Shenyang, Liaoning Province (LN; 124°02′ E, 42°07′ N): soil classification is loess-derived cinnamon soil (USDA soil taxonomy: Typic Hapludalfs), with a yellowish brown color and pH 5.7–7.0 (1:2.5 soil–water ratio); climatic conditions (growing season: April–September 2018) included a mean daily temperature of 20.4 °C and total precipitation of 569.8 mm. (2) Gongzhuling, Jilin Province (JL; 125°18′ E, 44°35′ N): soil classification is dark brown soil (USDA soil taxonomy: Typic Hapludolls), with a grayish brown to dark brown color and pH 6.0–7.1 (1:2.5 soil–water ratio); climatic conditions (growing season: April–September 2018) included a mean daily temperature of 18.7 °C and total precipitation of 543.1 mm. (3) Harbin, Heilongjiang Province (HLJ; 127°37′ E, 46°43′ N): soil classification is meadow soil (USDA soil taxonomy: Typic Haplaquolls), with a pale grayish yellowish brown color and pH 6.0 to 6.5 (1:2.5 soil–water ratio); climate conditions (growing season: April–September 2018) included a mean daily temperature of 18.1 °C, total precipitation of 639.5 mm. (Data sources: Soil properties from the Chinese Soil Database, http://vdb3.soil.csdb.cn/ accessed on 15 October 2025; meteorological data from the RP5 Weather Database, https://rp5.ru/ accessed on 16 October 2025; https://www.mirror-earth.com/ accessed on 16 October 2025). A randomized complete block design (RCBD) was adopted for the field experiment, with 4 blocks per site and 109 hybrid combinations assigned to each block (4 blocks × 109 combinations = 436 total combinations, consistent with the previously developed hybrids). At each of the three experimental locations (Shenyang, Gongzhuling, Harbin, China), a three-replicate RCBD was arranged, where each experimental unit was a double-row plot. Plot size and plant density were standardized: row length = 4 m, inter-row spacing = 65 cm, intra-row spacing = 25 cm, and 17 plants per row. Phenotypic data for 16 yield-related traits were collected at the physiological maturity stage of maze. The traits measured were plant height (PH), ear height (EH), stem diameter (SDR), stem length (SL), tassel branch number (TBN), tassel main axis length (TL), ear length (EL), ear diameter (ED), shaft diameter (SD), kernel row number (KRN), kernel number per row (KNPR), hundred kernel weight (HKW), kernel length (KL), kernel width (KW), grain thickness (GT), and yield per plant (YPP). A comprehensive phenotypic database for these 16 traits was established [34]. Phenotypic data were analyzed using R software (version 4.3.3; R Core Team, Vienna, Austria: R Foundation for Statistical Computing, 2023; https://www.r-project.org/ accessed on 16 February 2025) on the Windows 10 operating system. To minimize the confounding effects of environmental heterogeneity across the three sites, best linear unbiased predictions (BLUPs) for each phenotypic trait were calculated using the lmer function in the *lme4* package [35]. The processed phenotypic data were then utilized for subsequent analyses.

### 2.3. Genome-Wide Association Analysis

For the same set of Northeast China maize inbred lines (1970s–2010s) described in Section 2.1, leaf sampling was conducted at the three-leaf stage, with one unexpanded heart leaf collected per inbred line. DNA extraction obtained from maize grown under the same field conditions and locations described in Section 2.2 and used for the analysis of yield-related traits. Genomic DNA was extracted from the fresh tender leaf tissues using a modified cetyltrimethylammonium bromide (CTAB) method [36]. Qualified DNA samples were randomly sheared into 350 bp fragments using a Covaris S220 ultrasonic fragmentation system (Covaris, Woburn, MA, USA). The fragmented DNA was then processed through end-polishing, A-tailed, and ligated with full-length Illumina TruSeq adapters (Illumina, San Diego, CA, USA), followed by 12 cycles of PCR amplification to enrich adapter-ligated fragments. PCR amplicons were purified using AMPure XP magnetic beads (Beckman Coulter, Brea, CA, USA) to remove unincorporated primers and short fragments. Library quality assessment was conducted using an Agilent 2100 Bioanalyzer (Agilent Technologies, Santa Clara, CA, USA) for size distribution verification, and library concentration was quantified using real-time PCR (qPCR; KAPA Biosystems, Wilmington, MA, USA). Subsequently, the Illumina NovaSeq 6000 sequencing platform (Illumina, San Diego, CA, USA) was utilized to generate about 43.75 Tb of raw sequences with a 150 bp read length.

The remaining high-quality paired-end reads were mapped to the B73 reference genome (RefGen_v4) using Burrows-Wheeler Aligner (BWA) software [37] (Version 0.7.17) with the command ‘mem -t 4 -k 32 –M’. To reduce mismatches generated by PCR amplification before sequencing, duplicated reads were removed using the SAMtools (Version 0.1.1) [38]. After alignment, genomic variants for each inbred line were identified in Genome Variant Call Format (GVCF) using the Sentieon DNAseq software package Sentieon DNAseq software package (Sentieon, Inc., San Jose, CA, USA) [39]. Then, all GVCF files of all lines were merged into a single dataset. High-quality variants were filtered using the Genome Analysis Toolkit (GATK) software (Version 4.4.0.0) with the following criteria: QD > 2.0, FS < 60.0, MQ > 20.0, MQ Rank Sum > −12.5, and Read Pos Rank Sum > −8.0 [40]. Additionally, potential low-quality variants were removed based on the following filters: (1) two alleles only, (2) missing rate < 0.2, (3) minor allele individuals ≥ 5.0, (4) heterozygosity rate < 0.1, and (5) minor allele frequency ≤ 0.05. Consequently, 15 232 270 high-quality SNPs were identified. The identified SNPs were further annotated using ANNOVAR tool software (version 2013-05-20) [41]. Using phenotype data optimized based on GCA values from one year across three locations, GWAS was performed for yield-related traits. GWAS for the GCA of yield-related traits in the hybrid combination population was performed using the BLINK model in GAPIT software the GAPIT software (Version 3) [42]. The Bonferroni method was used to set the significance threshold at 3.5 × 10^−5^ (−log_10_ *p* > 6). SNPs were screened based on GWAS results from the GCA values and BLINK model conditions.

Using GWAS and resequencing data, based on the traits corresponding to GCA values that changed significantly during the breeding process, as well as the screened SNP information and genotypes of these traits, plotting analysis of the elite allele frequency was conducted using the ggplot2 package in R 4.3.3 [43].

### 2.4. Statistical Analysis

Combining ability analysis was performed using the NCII method proposed by Griffing [44]. GCA and SCA effects were calculated based on plot trait means.

Estimation of general combining ability (GCA) effects for testers:

$$Gcam=\frac{T_{m}}{rf}-\frac{T_{c}}{mfr}$$

Estimation of general combining ability (GCA) effects for tested lines:

$$Gcaf=\frac{T_{f}}{rm}-\frac{T_{a}}{mfr}$$

Estimation of specific combining ability (SCA) effects for tester × tested line combinations:

$$Scaij=\frac{T_{ij}}{r}-\frac{T_{f}}{rm}-\frac{T_{m}}{rf}+\frac{T_{c}}{mfr}$$

where f = number of tested lines under evaluation, r = number of replications, m = number of testers, j = the j-th female parent, and i = the i-th male parent.

The BLUP values for each trait in the association mapping hybrid combination population were calculated using MATE-R software, with the calculation based on the following formula:

$$Y_{ij}=\mu +Line_{i}+Env_{j}+{\left(Line\times Env\right)}_{ij}+{\left(Env\times Rep\right)}_{jn}+error_{ijn}$$

where

$Y_{ij}=$ best linear unbiased prediction (BLUP);

μ= grand mean;

$Line_{i}=$ phenotypic value of the i-th hybrid combination;

$Env_{j}=$ effect of the j-th environment;

${\left(Line\times Env\right)}_{ij}=$ phenotype–environment interaction effect;

${\left(Env\times Rep\right)}_{jn}=$ environment–replication interaction effect;

$error_{ijn}=$ error of the j-th environment and the n-th replication.

Data were processed and analyzed using SPSS 21.0, SAS 9.4, GraphPad Prism 10.0.2, Microsoft Excel, R 4.3.3, MATE-R, and other software.

## 3. Results

### 3.1. Analysis of Yield-Related Traits in Major Maize Inbred Lines from Northeast China with Tester PH4CV and PH6WC

Using PH4CV and PH6WC as testers, a combined analysis of variance (ANOVA) was performed on 16 yield-related traits of the testcross population, with the BLUP values of each plot as the unit. Detailed information on these 16 traits is provided in Table 1 and Table 2.

The results show that except for TBN and KRN, the other 14 traits exhibited significant variations (*p* < 0.05) across different locations. This indicates that geographical and ecological factors exerted a substantial impact on most traits under both tester backgrounds, suggesting that the ecological conditions of the experimental sites selected in this study differed notably, thus effectively meeting the requirements for multi-environmental testing. Regarding experimental blocks, significant differences (*p* < 0.05) were detected in TL, KL, KW, SD, SDR, and SL under both tester backgrounds. This implies that these traits were highly sensitive to block-specific environmental factors. Notably, under the PH4CV background, experimental blocks significantly affected EH and KL; in contrast, under the PH6WC background, such block effects were observed for ED and KNPR. These findings indicate divergent responses of trait to block environments under different tester genetic backgrounds. Among hybrid combinations, PH, EH, EL, and SD showed significant differences (*p* < 0.05) under both tester backgrounds, suggesting substantial phenotypic variation in these traits across test cross combinations. Specifically, under the PH4CV background, significant differences were observed in ED, KRN, KL, GT, SDR, SL, and YPP; whereas under the PH6WC background, significant differences were detected in KNPR, KW, and HKW. This highlights distinct genotypic responses of yield-related traits in test cross populations to different tester backgrounds. By contrast, the interactive effects of experimental blocks, geographical factors, and test cross combinations had minimal influence on all measured traits. Among the 436 test cross combinations, those with higher SCA effects for YPP included XF × PH6WC, PHPMO × PH4CV, 2511A × PH4CV, S121 × PH4CV, PHB1M × PH6WC, LD61 × PH6WC, Shen 151 × PH6WC, Liao 3162 × PH6WC, PH6JM × PH6WC, and Chengxi 60 × PH6WC. Among these, S121 × PH4CV exhibited the highest YPP_SCA_ effect, with a value of 0.56. Conversely, combinations with lower YPP_SCA_ effects included PHB1M × PH4CV, Longxi 53 × PH4CV, Ji846 × PH4CV, K12 × PH4CV, Liao8821 × PH4CV, KH786 × PH4CV, CA616 × PH4CV, Chengxi 53 × PH4CV, PHPMO × PH6WC, and S121 × PH6WC. Based on previous studies by our research group, both S121 and PHPMO belong to the PB heterotic group, while Shen 151 and LD61 belong to the PA heterotic group. This indicates that during maize breeding in Northeast China, most hybrid combinations between PB heterotic group inbred lines and PH6WC exhibit higher SCA; similarly, PH4CV combined with most inbred lines from the PA heterotic group also produces high SCA. Notably, the combinations that exhibited positive YPP_SCA_ effects while simultaneously showing positive SCA effects for all other ear and grain traits included Zhong 106 × PH4CV, z3-87 × PH4CV, M60 × PH4CV, F2001 × PH4CV, KW5G321 × PH6WC, and 9714 × PH6WC (Table S2). This suggests that in future breeding programs, when aiming to simultaneously improve YPP and maintain relatively good performance in other traits, priority should be given to combinations with high SCA effect values across the above-mentioned traits.

### 3.2. GCA Analysis of Major Maize Inbred Lines Traits in Northeast China

GCA analysis was conducted for 16 traits of 218 tested inbred lines and 2 testers across 436 test cross combinations from the Northeast China maize test cross population. The GCA effect values for each trait are presented in Table S1. Joint variance analysis (Table 3) showed that all traits exhibited significant (*p* < 0.05) or highly significant (*p* < 0.01) differences across environments, demonstrating that geographical environmental factors exerted substantial influences on the test cross population. For PH, EH, TL, KNPR, KL, KW, SDR, SL, and HKW, significant block-level environmental effects were observed among experimental blocks. These results suggest that most traits in the test cross population were notably sensitive to block-specific environmental conditions. Analysis for 218 tested inbred lines revealed significant (*p* < 0.05) or highly significant (*p* < 0.01) differences in GCA for 13 traits (excluding KNPR, KW, and SDR). This finding indicates substantial GCA variation and rich genetic diversity among the inbred lines for most of the measured traits. Additionally, highly significant differences in GCA for all traits were observed between the two testers, confirming that both testers were effective in meeting experimental requirements. Except for SDR, significant or highly significant interactions between tester GCA and environmental factors were detected for the remaining 15 traits, which implies that both tester GCA and environmental factors exerted strong influences on these traits. For the SCA of hybrid combinations (Tests and Lines), significant or highly significant differences were observed for 15 traits (excluding KRN), reflecting substantial phenotypic variation among the test cross combinations. Notably, highly significant interactions between SCA (Tests and Lines) and environment were identified for 11 traits (excluding TBN, KRN, KW, SDR, and SL). These results suggest that SCA (Tests and Lines)–environment interactions had significant effects on most traits in the test cross population.

For PH, among the 218 tested inbred lines, 117 showed negative GCA effects, while 101 exhibited positive GCA effects. Notably, the inbred line Xingkenzi 101-1 had the highest GCA effect value at −47.4111. In terms of TL, line 391 demonstrated the highest GCA effect value of 5.40244, with 111 inbred lines showing positive GCA effects. For TBN, 110 tested lines showed positive GCA effects, with Aijin 525 having the highest effect value of 8.71. For KL, 114 tested lines showed positive GCA effects, with LD61 having the highest effect value of 1.34. Regarding EL, 116 inbred lines showed positive GCA effects, with Zhongzi 01 having the highest GCA effect value of 3.46934. For the SDR trait, 102 tested lines exhibited positive GCA effects, among which M502 had the highest effect value (−3.09148). For HKW, 109 tested lines showed positive GCA effects, with Huang C having the highest effect value of 9.20. In terms of YPP, 103 tested lines showed positive GCA effects, with Dan 598 having the highest effect value of 0.44. GCA effect analysis of the 218 Northeast China inbred lines identified the top 10 materials with high YPP GCA as Dan 99 Chang, M5972, Tie T0403, Chang 7-2, Xin 444, Jing 92, 7884-Ht, Dan 598, TM, and 7-61. Specifically, Chang 7-2 exhibited strong GCA effects for both YPP and TBN, indicating its potential to enhance yield while improving tassel structure. Jing 92 showed high YPPGCA and KWGCA, along with positive GCA effects for EL, ED, TBN, KRN, KNPR, KL, SL, and HKW. This suggests Jing 92 can not only increase YPP but also drive improvements in multiple ear-related traits during breeding. Dan 598 displayed significant YPPGCA, alongside high GCA effects for ED, TBN, and KL. Additionally, it showed positive GCA for EL, TL, KRN, KNPR, KW, and SL, highlighting its role in simultaneously enhancing yield and improving both tassel and ear traits.

### 3.3. Trends of GCA and Correlations for Yield-Related Traits

**Figure 1:**
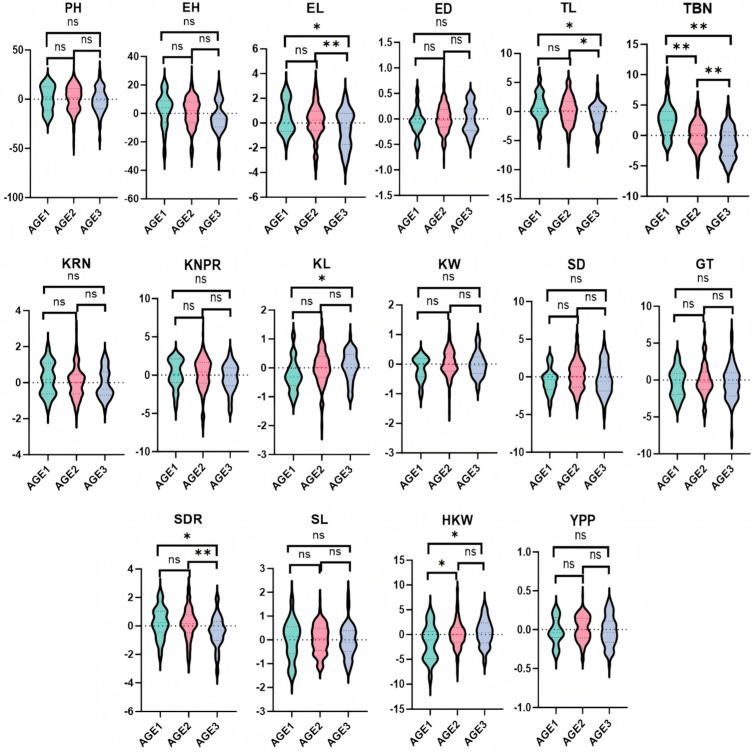
GCA of 16 yield traits at different breeding stages. Plant height (PH), ear height (EH), stem diameter (SDR), stem length (SL), tassel branch number (TBN), tassel main axis length (TL), ear length (EL), ear diameter (ED), shaft diameter (SD), kernel row number (KRN), kernel number per row (KNPR), hundred kernel weight (HKW), kernel length (KL), kernel width (KW), grain thickness (GT), and yield per plant (YPP). ns denotes no significant difference, * denotes a significant difference(*p* < 0.05), ** denotes an extremely significant difference level (*p* < 0.01).

As shown in Figure 1, in the test cross population of Northeast China maize, the GCA for PH and EH exhibited a gradual decreasing trend with the advancement of the breeding process. Overall, the responses of PH_GCA_ and EH_GCA_ effects to breeding selection did not show significant increases, while remaining positively effective. For EL and ED, their GCA values showed a trend of first increasing and then decreasing with breeding progression. Specifically, EL_GCA_ reached an extremely significant difference level (*p* < 0.01) in the AGE3, with its mean value decreasing from 0.13 in AGE2 to −0.48 in AGE3. Notably, EL_GCA_ decreased significantly in AGE3, indicating a distinct negative response to breeding selection, whereas ED_GCA_ showed no significant decrease. The GCA for TL and TBN showed a continuous decreasing trend. TL_GCA_ reached a significant difference level (*p* < 0.05) in AGE3, with its mean value decreasing from 0.15 in AGE2 to −0.62 in AGE3. TBN_GCA_ showed extremely significant differences (*p* < 0.01) across AGE1-AGE3. The mean TBN_GCA_ value decreased from 2.51 in AGE1 to −1.28 in AGE3. The obvious decreases in TL_GCA_ at AGE3 and TBN_GCA_ throughout all stages suggested clear responses of these traits to breeding selection. For KRN, a gradual decrease in GCA was observed, while the GCA of KNPR remained basically stable, indicating no significant reduction in their responses to breeding selection. KL_GCA_ showed a gradual increase, with significant differences (*p* < 0.05) across AGE1-AGE3, reflecting a positive and obvious response to breeding selection. Meanwhile, KW_GCA_ remained stable without significant reduction. SD_GCA_ first increased and then decreased, with no obvious upward trend in its response to breeding selection. GT_GCA_ remained stable, indicating no significant reduction in its response. Both SDR_GCA_ and stalk length SL_GCA_ showed gradual decreases, with SDR_GCA_ reaching an extremely significant level (*p* < 0.01) in AGE3. The mean SDR_GCA_ value decreased from 0.13 in AGE2 to −0.41 in AGE3. The significant decrease in SDR_GCA_ in AGE3 indicates a clear positive response to breeding selection. In contrast, SL_GCA_ showed no obvious increasing trend. HKW_GCA_ first increased and then stabilized, with significant differences (*p* < 0.05) observed in AGE2. The obvious increase in HKW_GCA_ in AGE2 suggests a distinct positive response to breeding selection. Similarly, YPP_GCA_ first increased and then stabilized, showing no significant reduction throughout the breeding process.

**Figure 2:**
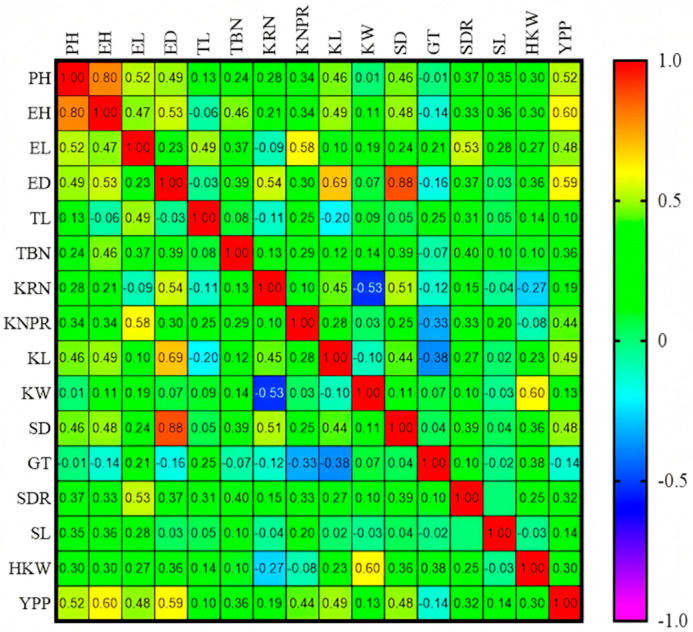
Correlation analysis of GCA of 16 yield traits in Northeast China. Plant height (PH), ear height (EH), stem diameter (SDR), stem length (SL), tassel branch number (TBN), tassel main axis length (TL), ear length (EL), ear diameter (ED), shaft diameter (SD), kernel row number (KRN), kernel number per row (KNPR), hundred kernel weight (HKW), kernel length (KL), kernel width (KW), grain thickness (GT), and yield per plant (YPP).

Correlation analysis of GCA among major maize inbred lines from Northeast China (Figure 2) showed that PH_GCA_ was positively correlated with EH_GCA_ (*r* = 0.80). ED_GCA_ exhibited a strong positive correlation with SD_GCA_ (*r* = 0.88). Additionally, KL_GCA_ was also positively correlated with SD_GCA_. YPP_GCA_ was positively correlated with the GCA values of EL, ED, KRN, KNPR, and HKW. These results confirm the high stability and accuracy of the aforementioned GCA effects, indicating that the observed genetic associations among traits are reliable.

### 3.4. GWAS for GCA of Yield-Related Traits in Maize from Northeast China

**Figure 3:**
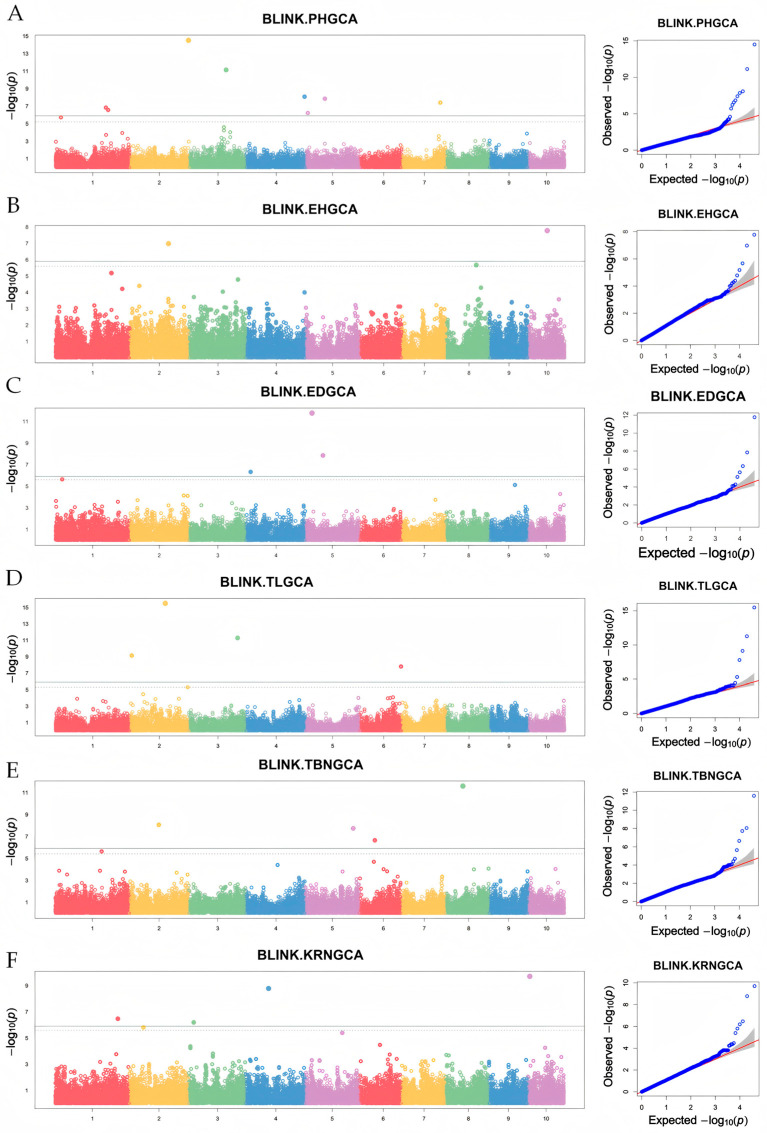
Genome-wide association analysis of GCA of maize populations in Northeast China. (**A**) PH; (**B**) EH; (**C**) ED; (**D**) TL; (**E**) TBN; (**F**) KRN. BLINK (Bayesian-information and Linkage-disequilibrium Iteratively Nested Keyway) is a highly efficient multi-locus GWAS model that is suitable for large-scale genomic data analysis. The left graph is a Manhattan plot; the right is a QQ plot.

In this study, genotype data of inbred lines resequencing were used to perform GWAS on the values of GCA traits in Northeast China maize test cross populations based on the BLINK model. The experimental data conformed to a normal distribution. Significant markers were identified using the threshold of 3.5 × 10^−5^ (i.e., −log_10_ *p* > 6) (Figure 3 and Figure S1). A total of 38 loci were identified across 12 traits using the BLINK model, and the results, as indicated by Manhattan plots and QQ plots, are detailed as follows: PH: eight SNPs located on chromosomes 1, 2, 3, 4, 5, and 7; EH: two SNPs on chromosomes 2 and 10; ED: three SNPs on chromosomes 4 and 5; TL: four SNPs on chromosomes 2, 3, and 6; TBN: four SNPs on chromosomes 2, 5, 6, and 8; KRN: four SNPs on chromosomes 1, 3, 4, and 10; KW: one SNP on chromosome 10; SD: six SNPs on chromosomes 2, 3, 5, 6, and 8; GT: two SNPs on chromosomes 2 and 10; SDR: two SNPs on chromosomes 3 and 8; SL: one SNP on chromosome 8; HKW: one SNP on chromosome 8. For the PH trait, half of the associated markers were predominantly distributed on chromosomes 1 and 5, with the markers S1-217817403 on chromosome 1 and S5-9690315 on chromosome 5 exhibiting high minor allele frequencies (MAFs). For the ED trait, the SNP marker S5-27726473 had the highest MAF value of 0.3560976. For the TL trait, the SNP markers S3-201614406 and S6-171551982 showed relatively high MAF values of 0.4317073 and 0.4268292, respectively. Among the markers associated with TBN, the highest MAF was 0.3731707. The SNP associated with the KRN trait had the highest MAF value of 0.4682927, located on chromosome 3. For the SD trait, the highest MAF value of 0.4048780 among associated SNPs was observed on chromosome 8. The marker for the HKW trait exhibited a MAF value of 0.2219512. The marker with the highest overall MAF across all SNPs was S1-217817403 on chromosome 1. Notably, a SNP (S5-27726473) on chromosome 5 was found to be associated with both SD and ED, indicating the presence of pleiotropy for the locus (Table 4).

### 3.5. Trends in Elite Alleles of GCA During Breeding Stage

**Figure 4:**
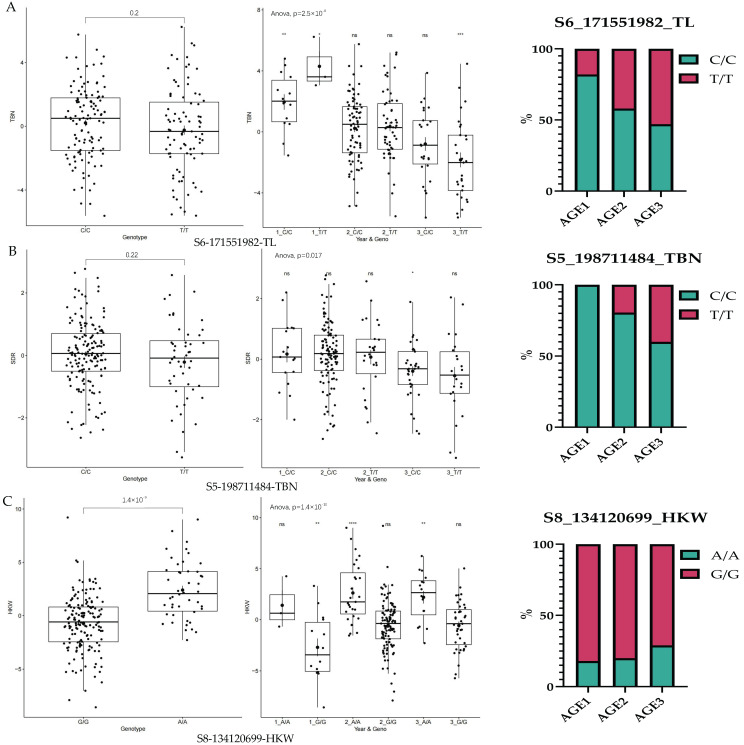
Trends of elite alleles for GCA during the breeding stage. (**A**) TL; (**B**) TBN; (**C**) HKW. ns denotes no significant difference, * denotes a significant difference, ** denotes a highly significant difference, *** denotes a very highly significant difference, **** denotes an extremely highly significant difference.

Elite alleles were analyzed based on the significance of GCA differences in Northeast China maize populations during modern breeding and GWAS (Figure 4, Figures S2 and S3). The key findings are as follows: For the TL trait, at SNP marker S2-8474932, two alleles were identified, with T/T predominant and C/C less frequent. At SNP marker S2-147092451, alleles were evenly distributed; G/G showed higher GCA in early-to-middle breeding stages, while T/T exhibited higher GCA in late breeding stages. At SNP locus S3-201614406, alleles were evenly distributed, with T/T showing higher GCA. At SNP locus S6-171551982, two alleles were evenly distributed. For the TBN trait, at S2-121462400, C/C was the predominant allele, with higher GCA throughout the entire breeding process. At S5-198711484, C/C was more frequent than T/T; only C/C was present in early breeding stage, while both T/T and C/C appeared in middle-to-late breeding stages. At S6-64593869, C/C was predominant, but T/T showed higher GCA across all breeding stages. For the HKW trait, G/G was the predominant allele, but A/A showed higher GCA across all breeding stages, indicating its potential to enhance HKWGCA in inbred lines. The GCA marker locus S5-198711484 showed only one allele (C/C) in early breeding stages. A total of four TL elite alleles were found to reduce TLGCA during the breeding process. The A/A elite alleles at S8-134120699 for HKW showed higher GCA, serving as a reference for future maize breeding strategies (Figure 3, Figure 4, Figures S2 and S3).

## 4. Discussion

The northeastern maize region is one of the most important maize production areas in China [2]. In this study, we found a significant increase in KL_GCA_ and HKW_GCA_ by using diverse maize germplasm resources from different breeding stages in Northeast China. This finding indicates that maize inbred lines in Northeast China exhibit high efficiency in utilizing genetic bases and favorable alleles to improve KL and HKW. Yu et al. [45] analyzed the combining ability of maize germplasm across different ecotypes worldwide and found that inter-ecotype crosses (e.g., temperate × tropical) could significantly enhance heterosis, with particularly notable effects on EL and ED. In this study, EL_GCA_ exhibited distinct dynamic trends during the breeding process, which is consistent with previous findings. However, differences were also observed; this study focused on germplasm resources from different breeding stages in Northeast China, where tropical maize germplasm is relatively scarce. Consequently, the dynamic trends of ED_GCA_ during the breeding process may differ from previous reports, indicating that the breeding objectives may vary across ecological regions.

Zhang et al. [46] employed the NCII design and selected 4 tester lines and 125 inbred lines to develop hybrid combinations. Their study on the GCA effects of yield traits showed that 13 inbred lines exhibited high GCA for yield, and these lines also demonstrated excellent performance in the GCA of related traits. Wang et al. [47] employed the NCII design to evaluate the combining ability of 14 elite inbred lines. Their results revealed that the inbred line Zhong Q897-2 exhibited the highest GCA. Hybrid combinations derived from Zhong Q897-2 demonstrated desirable agronomic traits, including low ear height and high 100-kernel weight (HKW). Li et al. [48] analyzed 14 phenotypic traits of 326 inbred lines and found that Group I included 146 germplasms, primarily characterized by a dwarf plant architecture, short tassels with few branches, and small ear size. In this study, GCA analysis of 218 maize inbred lines showed that TBN_GCA_ decreased significantly, while HKW_GCA_ and KL_GCA_ increased significantly across the overall breeding stage. These results are consistent with previous research findings, indicating that breeders’ breeding objectives remained consistent during the breeding stage. This study utilized the NCII genetic mating design with the parental lines of PH4CV and PH6WC as testers, where PH4CV serves as a key inbred line representing the Reid heterotic group and PH6WC represents the Lancaster heterotic group. Analysis of yield-related traits in 436 test cross combinations revealed that KL and HKW increased significantly in the AGE2 among test cross combinations derived from major Northeast China maize inbred lines. These results indicate that breeders prioritized grain trait selection responses during the transition from AGE1 to AGE2, which is consistent with previous findings. TBN and SDR decreased significantly in the AGE3, suggesting that breeders have increasingly focused on selecting materials with fewer TBNs and thinner SDRs under natural selection pressures in modern breeding. This trend may be attributed to the lodging resistance advantages conferred by thinner SDRs, which aligns with earlier research findings.

The accumulation of favorable allele frequencies influences GCA effects. Imdad et al. [49] calculated GCA and performed a GWAS to identify polymorphic SNPs in 33 hybrid rice lines. Their results indicated that parental lines carrying elite alleles could serve as superior breeding parents, enabling molecular design breeding based on GCA-associated alleles in parental genomes. Chen et al. [50] revealed the genetic basis of combining ability in rice through association analysis and found that favorable GCA alleles (Ghd8, GS3, and qSSR4) accumulate in high-GCA parental lines, explaining 30.03% of the phenotypic variation in GCA for grain yield—demonstrating the feasibility of molecular breeding for developing high-GCA parental lines. In this study, we found that for HKW, the GCA of the G/G elite alleles with a higher frequency of favorable alleles was significantly higher than that of the A/A alleles, with more in AGE3. This observation could be attributed to the accumulation of elite alleles, which is consistent with previous findings. Additionally, we observed that the T/T alleles for TBN was absent in AGE1 but gradually appeared in AGE2 and AGE3, representing a transition from “absence” to “presence” across breeding stages. Concurrently, both TBN_GCA_ and the trait itself (unpublished data) decreased during the breeding process. This result demonstrates that the variation trend of GCA is associated with the accumulation of favorable allele frequencies, which aligns with findings from previous studies.

Breeding selection is a key factor shaping the maize breeding process. This study explored the dynamic trends of GCA during the breeding process, as GCA variation is driven by the response of traits to breeding selection. Although not all traits exhibit significant trends in GCA over the breeding process, analysis of Figure 1 shows that if EH and KRN exhibit significant differences in the next breeding stage, it will indicate that breeders are paying relatively close attention to the response of EH and KRN to GCA selection. Based on the current analysis results, the GCA of EL, TL, TBN, KL, SDR, and HKW are all affected by their response to breeding selection. This indicates that the aforementioned traits receive greater attention from breeders and may also serve as core targets for future breeding programs. Meanwhile, we observed that for the TL trait across all experimental materials, the T/T genotype in line S2-8474932 gradually increased with breeding progression, while the C/C genotype gradually decreased. This phenomenon suggests that breeding materials carrying the T/T genotype are more favored by breeders. In contrast, the C/C genotype is declining under breeding selection pressure and may eventually be eliminated in future breeding generations. Additionally, future methods to improve TL general combining ability (TL_GCA_) may involve increasing the frequency of the T allele, which is associated with the T/T genotype. Furthermore, the A/A genotype at locus S8-134120699 (linked to hundred-kernel weight, HKW) exhibits higher GCA. This genotype can effectively improve HKW_GCA_ in future maize breeding, which is particularly important for increasing yield. In this study, using the BLINK model, a total of 38 significantly associated loci were identified. A 50 kb region upstream and downstream of each significant locus was designated as the candidate interval. By integrating the significant differences in GCA of Northeast China maize populations across breeding stages, a total of 11 candidate genes were screened and functionally annotated. Among these, more functionally annotated candidate genes were identified for TL. Specifically, the protein encoded by the Homeobox gene (*Zm00001d043484*) functions as a transcription factor, which plays a crucial role in regulating the ontogeny and cell differentiation of eukaryotes. It regulates gene expression by binding to DNA, thereby influencing processes such as plant morphogenesis and organ development. These findings provide a theoretical basis for future studies on maize tassel length (TL).

Inbred lines with high GCA are critical for developing superior maize hybrids. Li et al. [51] collected 1604 maize inbred lines, conducted phenotypic evaluation and genome sequencing, and identified dynamic trends of agronomic traits in female heterotic groups (FHGs) and male heterotic groups (MHGs) during the breeding process. Among these traits, TBN showed a significant downward trend in both FHGs and MHGs throughout the breeding period. Wang et al. [9] performed phenotypic evaluation and GWAS on 350 maize inbred lines from multiple breeding eras in China and the United States, and found that TBN exhibited a marked downward trend during breeding. Our research group previously conducted phenotypic evaluation and resequencing on major inbred lines from the Northeast China maize region and also observed a downward trend in TBN during breeding (unpublished data). Focusing on GCA in this study, we detected a significant downward trend in TBN_GCA_ during the breeding process. Since GCA values are estimated from phenotypic data, it can be inferred that the variation trend of GCA may be consist with that of the TBN phenotypic trait itself. Previous studies have demonstrated that phenotypic traits and their corresponding GCA effect values share the same set of genetic loci [52,53]. Future research could involve QTL analysis and candidate gene mining for GCA of each target trait, which would more effectively enhance the application of GCA effects in maize inbred line selection and breeding.

## 5. Conclusions

This study aimed to characterize the dynamic trends of combining ability in major maize inbred lines from Northeast China during the 1970s–2010s and identify genetic loci associated with GCA via a GWAS. The results show that the GCA of six yield traits exhibited significant responses to breeding selection. The mean TBN_GCA_ value decreased from 2.51 in AGE1 to −1.28 in AGE3, indicating a negative response to selection. TL was classified into four elite alleles, whereas TBN was associated with three elite alleles. Among these, the three TBN-associated elite alleles showed a decreasing trend in GCA during the breeding process, Notably, in the early breeding stage, TBN was predominantly controlled by the C/C elite alleles during AGE1, and the A/A alleles of the HKW exhibited higher GCA. These findings provide valuable references for enhancing HKW_GCA_ and exploring the breeding stage trends of TBN in future maize breeding programs.

## Figures and Tables

**Table 1:** Joint variance analysis of 16 yield-related traits under the PH4CV tester background.

Traits	Blo	Loc	Cro	Blo × Loc	Blo × Cro	Loc × Cro	Blo × Loc × Cro
DF	3	2	217	6	651	434	1302
PH	431.93	170,653.76 **	1935.44 *	205.38	181.73	314.35	155.10
EH	386.79 *	45,063.38 **	1182.96 **	426.61 **	80.10	106.09	78.52
EL	25.05	56.27 *	23.44 *	3.78	3.91	4.47	3.51
ED	0.03	2.02 **	0.59 **	0.34 *	0.09	0.14	0.09
TL	120.97 *	1724.24 **	58.43	44.38	9.38	11.32	7.77
TBN	10.42	78.74	42.48	30.84	6.10	6.75	5.86
KRN	1.36	6.26 *	5.40 *	0.14	1.86	2.91	1.36
KNPR	75.67	1116.03 **	40.12	200.56 *	19.58	33.47	18.61
KL	3.45 *	13.67 **	2.73 *	1.95	0.42	0.62	0.38
KW	62.86 **	84.56 **	1.55	55.82	0.54 **	0.94	0.74
SD	62.25 **	949.66 **	27.96 *	84.11 **	3.86	4.48	3.35
GT	15.45	870.07 **	50.81 *	46.66 *	7.64	15.71	7.90
SDR	20.59 **	4370.51 **	14.82 **	103.28 **	6.11 *	5.40	4.74
SL	164.35 **	1034.33 **	4.77	114.14 **	2.13	2.84	2.26
HKW	6.22	1440.90 **	65.85	8.62	7.80	16.02	7.62
YPP	0.08	0.93 **	0.34 *	0.10	0.07	0.08	0.05

Note: The data in the table are the mean square values (*MS*); * and ** indicate respectively significant differences at the 0.05 and 0.01 levels. Blo represents blocks; Loc represents location, Cro represents cross, and DF represents degrees of freedom.

**Table 2:** Joint variance analysis of 16 yield-related traits under the PH6WC tester background.

Traits	Blo	Loc	Cro	Blo × Loc	Blo × Cro	Loc × Cro	Blo × Loc × Cro
DF	3	2	217	6	651	434	1302
PH	1180.91	131,570.40 **	1862.35 *	115.34	167.99	335.17	128.80
EH	79.21	29,114.31 **	1060.37 **	842.73 *	81.17	102.29	69.36
EL	3.79	26.86 *	18.44 *	4.03	2.04	3.40	2.17
ED	0.18	2.79 *	0.64	0.33	0.07	0.09	0.06
TL	104.25 *	1814.83 **	51.73	38.85	7.53	8.12	6.17
TBN	24.81	45.00	77.70	34.84	5.70	7.99	7.02
KRN	5.57	2.35	6.67	2.83	1.79	3.26	1.64
KNPR	133.93	122.37 *	50.85 *	99.99	20.14	38.46	22.38
KL	1.02	37.20 *	2.99	0.30	0.45	0.76	0.43
KW	88.05 **	115.81 **	1.98 *	72.78 **	0.65	0.92	0.83
SD	52.08 *	408.12 **	32.90 *	105.91 **	4.18	5.59	3.91
GT	11.53	153.31 *	34.83	32.19	6.46	10.29	5.82
SDR	23.45 *	4229.27 **	12.68	141.79 **	4.06	3.65	3.83
SL	84.67 **	994.61 **	4.18	78.70 **	1.72	2.78	1.95
HKW	22.10	2112.79 **	75.27 *	16.99	7.69	18.79	8.21
YPP	0.05	1.67 **	0.27	0.07	0.05	0.07	0.04

Note: The data in the table are the mean square values (*MS*); * and ** indicate respectively significant differences at the 0.05 and 0.01 levels. Blo represents blocks, Loc represents location, Cro represents cross, and DF represents degrees of freedom.

**Table 3:** Joint variance analysis of combining ability for yield-related traits in Northeast China.

Traits	Loc	Blo	Line	Line × loc	Tester	Tester × Loc	Line × Tester	Line × Tester × Loc
DF	2	3	217	434	1	2	217	434
PH	288,784.40 **	1308.83 **	15,899.80 **	1288.49 **	2914.74 **	450.09 **	807.09 **	198.51 **
EH	69,987.21 **	315.91 *	416.74 *	839.93 **	1916.82 **	116.10 **	268.90 **	88.66 *
EL	15.47 **	7.97	321.93 **	97.97 **	27.98 **	4.87 **	12.57 **	3.39 *
ED	7.36 **	0.02	1.41 **	1.19 **	1.01 **	0.13 **	0.19 **	0.11 **
TL	3356.62 **	227.59 **	463.13 **	11.93	85.38 **	10.22 **	22.63 **	9.48 **
TBN	119.97 **	4.65	5418.17 **	8.88	102.96 **	8.49 **	15.27 **	6.61
KRN	6.03 *	4.42	5.16	7.00 *	11.11 **	4.52 **	1.66	1.72
KNPR	1113.90 **	324.60 **	7065.20 **	820.96 **	58.76 **	45.77 **	29.58 **	25.05 **
KL	42.87 **	2.91 **	134.02 **	7.93 **	4.23 **	0.93 **	1.24 **	0.52 **
KW	398.90 **	119.45 **	1.45	0.1	2.81 **	1.18 *	1.12	0.9
SD	1231.03 **	9.8	29.17 **	62.15 **	52.22 **	5.58 **	7.70 **	5.10 **
GT	883.52 **	3.3	18,593.25 **	228.38 **	66.30 **	17.59 **	18.35 **	11.96 **
SDR	8397.87 **	17.25 *	0.82	0.15	20.24 **	5.09	6.90 **	3.95
SL	1787.86 **	246.36 **	95.58 **	51.05 **	6.20 **	3.20 **	2.85 *	2.47
HKW	3841.97 **	31.95 *	8472.96 **	42.48 **	108.12 **	25.30 **	24.34 **	11.24 **
YPP	3.62 **	0.09	2.72 **	0.14	0.40 **	0.09 **	0.19 **	0.07 **

Note: The data in the table are the mean square values (*MS*); * and ** indicate significant differences at the 0.05 and 0.01 levels, respectively. Blo represents blocks, Loc represents location, and DF represents degrees of freedom.

**Table 4:** SNP information of the GCA of the main traits in Northeast China maize populations.

Trait	SNPs	Chromosome	Position (bp)	*p* Value	MAF
PH	S1_208497876	1	208497876	1.459878 × 10^−7^	0.0926829
PH	S1_217817403	1	217817403	2.732274 × 10^−7^	0.4731707
PH	S2_242867385	2	242867385	3.079791 × 10^−15^	0.0658536
PH	S3_154176114	3	154176114	7.300432 × 10^−12^	0.1560975
PH	S4_242979954	4	242979954	8.332607 × 10^−9^	0.2707317
PH	S5_9690315	5	9690315	6.014419 × 10^−7^	0.4341463
PH	S5_80232841	5	80232841	1.405806 × 10^−8^	0.2463414
PH	S7_160382018	7	160382018	3.959368 × 10^−8^	0.2146341
EH	S2_160298536	2	160298536	1.038463 × 10^−7^	0.0902439
EH	S10_79122567	10	79122567	1.669485 × 10^−8^	0.0487804
ED	S4_20634817	4	20634817	4.724012 × 10^−7^	0.3390244
ED	S5_27726473	5	27726473	1.693935 × 10^−12^	0.3560976
ED	S5_72936373	5	72936373	1.417658 × 10^−8^	0.3292683
TL	S2_8474932	2	8474932	7.390562 × 10^−10^	0.0878048
TL	S2_147092451	2	147092451	3.235304 × 10^−16^	0.3975609
TL	S3_201614406	3	201614406	5.281157 × 10^−12^	0.4317073
TL	S6_171551982	6	171551982	1.589827 × 10^−8^	0.4268292
TBN	S2_121462400	2	121462400	8.794725 × 10^−9^	0.3731707
TBN	S5_198711484	5	198711484	1.853238 × 10^−8^	0.2414634
TBN	S6_64593869	6	64593869	2.240302 × 10^−7^	0.2926829
KRN	S1_259340649	1	259340649	3.391704 × 10^−7^	0.3536585
KRN	S3_21754072	3	21754072	6.418560 × 10^−7^	0.4682927
KRN	S4_95867722	4	95867722	1.648411 × 10^−9^	0.1609756
KRN	S10_9329687	10	9329687	1.973185 × 10^−10^	0.3975610
KW	S10_91130327	10	91130327	1.104042 × 10^−7^	0.3487805
SD	S2_102852731	2	102852731	1.045193 × 10^−7^	0.0756097
SD	S2_150753314	2	150753314	2.675444 × 10^−7^	0.2951219
SD	S3_199474950	3	199474950	5.598493 × 10^−7^	0.1853658
SD	S5_27726473	5	27726473	3.455339 × 10^−10^	0.3560975
SD	S6_87803583	6	87803583	6.698875 × 10^−15^	0.1756097
SD	S8_157428343	8	157428343	1.370149 × 10^−9^	0.4048780
GT	S2_71773878	2	71773878	2.280263 × 10^−10^	0.1560976
GT	S10_6348231	10	6348231	5.224577 × 10^−7^	0.0902439
SDR	S3_124027250	3	124027250	1.641799 × 10^−8^	0.1243902
SDR	S8_115429920	8	115429920	6.130413 × 10^−7^	0.0585365
SL	S8_105193919	8	105193919	4.301483 × 10^−11^	0.2682927
HKW	S8_134120699	8	134120699	2.863774 × 10^−14^	0.2219512

## Data Availability

All other relevant data and SAS codes are available from the corresponding authors upon request.

## Supplementary Materials

The following supporting information can be downloaded at https://www.mdpi.com/article/10.3390/cimb47110877/s1.
