# Supplementary material for: Genetic Trends in General Combining Ability for Maize Yield-Related Traits in Northeast China
Source: Curr Issues Mol Biol. 2025 Oct 23;47(11):877. doi: 10.3390/cimb47110877 (PMC12651405; doi:10.3390/cimb47110877)
Supplement: Supplementary file 1 [file cimb-47-00877-s001.zip › cimb-3888242-supplementary.pdf]

Table S1. GCA data of major maize inbred lines in Northeast China

Line	PH	EH	EL	ED	TL	TBN	KRN	KNPR	KL	KW	SD	GT	SDR	SL	HKW	YPP
Dong46	-5.21	3.38	-0.74	-0.07	1.04	1.78	0.37	-2.65	-1.15	-0.22	1.58	0.73	-2.00	0.55	-4.52	-0.04
A801	17.59	4.12	0.69	-0.04	1.26	-0.88	0.05	1.26	0.65	0.31	0.54	1.68	0.79	0.69	0.48	-0.08
Liao68	-1.66	0.78	0.91	-0.25	0.93	0.50	-0.32	2.62	-0.48	-0.47	-1.41	-1.45	0.56	0.06	-3.32	0.00
LIao3180	13.90	-2.26	0.59	-0.03	5.08	-3.91	0.54	-0.07	-0.73	-0.22	0.90	6.42	0.11	0.72	0.17	0.00
K12	15.47	10.09	2.50	0.41	3.55	0.59	1.23	2.53	-0.27	0.08	5.36	6.55	0.79	-0.35	6.92	0.06
Shen151	-3.54	-5.41	0.35	0.18	1.41	1.55	0.06	1.00	0.42	0.07	2.02	0.27	2.57	-0.81	-0.14	-0.17
Dan717	4.50	2.24	1.66	-0.05	3.75	-1.49	-0.50	2.36	-0.14	-0.05	0.07	0.91	0.99	-0.06	0.80	0.13
Liao8478	7.38	3.66	0.84	0.00	0.57	1.43	-0.28	1.83	0.25	-0.12	0.26	0.40	0.64	-0.43	-1.28	0.06
Zheng22	-6.44	-11.37	1.39	-0.19	1.84	0.29	-0.88	1.09	-0.27	0.07	-2.11	0.26	-0.35	-1.03	-1.16	0.18
Zhong106	0.68	12.32	-0.69	0.09	-1.12	1.90	-1.96	-1.96	0.20	1.00	0.67	0.49	-0.15	-0.09	7.92	0.14
Liao3162	-10.38	-11.08	-1.27	-0.08	0.13	-1.71	0.92	-5.51	-0.25	-0.62	-0.32	3.61	-0.55	-0.17	0.72	-0.15
Dong237	-17.11	-16.73	0.48	-0.41	-0.46	0.51	1.08	0.17	-0.94	-1.04	-2.71	-1.94	-0.03	-1.30	-8.58	-0.09
PH09B	-10.92	-12.28	-0.68	-0.28	0.67	-4.85	-0.57	-5.44	0.02	0.10	-2.80	2.11	-1.58	0.22	-1.34	0.01
PHPMO	-15.21	-11.05	-3.05	-0.40	-2.48	-4.03	0.38	-3.31	-0.56	-0.34	-2.45	-0.45	-1.58	-0.75	-2.49	-0.07
Dan988	12.34	6.43	1.38	0.01	1.70	3.09	-0.55	1.18	-0.54	0.41	1.79	0.66	0.23	-0.36	-0.20	0.15
DanT138	11.39	6.25	1.06	0.27	2.38	0.14	0.16	1.45	1.03	0.02	0.90	-0.33	2.05	-0.24	3.05	0.19
Liao6082	10.45	12.30	0.73	0.25	0.64	-0.32	0.41	2.64	0.85	-0.02	0.05	-4.22	0.97	0.79	-2.18	0.14
63792	1.16	0.05	-0.86	0.15	0.27	-2.80	-0.30	0.01	0.85	-0.13	-0.45	-1.29	-0.97	0.40	2.50	-0.02
7017	-1.97	-1.82	1.98	-0.13	3.03	-2.49	-0.56	1.70	-0.52	1.00	-0.65	0.64	-0.05	-0.68	1.75	0.14
Dan99C	14.28	15.39	2.35	0.21	0.76	-0.83	0.71	2.00	0.50	-0.31	2.86	1.59	1.21	0.34	1.71	0.27
Shen3336	-2.64	7.38	-0.25	0.05	-4.37	-1.40	0.16	-1.72	-0.15	-0.10	-0.01	-0.27	0.08	0.05	0.89	0.05
Shen3265	10.89	12.54	1.29	0.41	-0.99	0.61	0.74	1.09	-0.12	-0.07	2.77	0.65	0.40	-0.15	1.45	0.23
Hai268	-18.62	2.55	-0.27	0.23	-1.46	2.52	1.35	0.36	0.06	-0.47	0.75	-0.93	-0.98	0.06	-3.24	-0.08
T98103_1	0.67	-10.46	0.30	-0.14	1.69	-0.88	1.39	1.63	-0.23	-0.03	-1.35	-0.27	-1.63	0.44	-2.98	-0.20
Dan360	12.80	11.36	1.08	0.70	0.19	2.83	1.92	-1.25	1.19	-0.63	3.84	-0.05	1.30	0.56	0.89	0.24
CL143	2.93	6.85	0.96	0.10	-1.94	0.28	0.53	-1.11	-0.01	0.68	1.83	4.27	1.33	0.79	2.64	-0.04
CH382	15.86	11.65	3.15	0.33	0.69	1.28	1.07	1.00	0.01	-0.48	3.13	4.02	1.86	0.38	1.56	0.15
PH6JM	8.96	7.93	-0.84	-0.17	-0.19	-1.95	-0.61	-0.13	0.19	0.29	-2.50	0.80	-0.76	-0.11	1.63	-0.09
Dan6263	24.90	5.75	2.41	0.06	2.84	-0.79	0.28	3.75	0.43	-0.27	-0.09	0.05	1.50	0.33	-0.58	0.18
C260	16.15	4.12	0.65	-0.03	-0.56	-0.67	0.52	-1.25	0.24	0.25	-0.52	-0.83	1.54	-0.61	-1.06	-0.26
C168	11.59	11.75	-1.00	0.38	-1.13	5.20	0.41	0.54	0.10	-0.16	2.45	-2.16	0.72	-0.13	0.67	0.10
Liao7980	7.58	9.77	-0.21	0.29	-2.98	-0.04	0.09	0.37	0.82	0.38	1.48	-1.96	0.01	0.65	0.22	0.10
F12	15.73	5.68	0.18	0.43	-1.76	-0.46	1.31	1.41	0.94	-0.42	2.76	-1.11	-0.09	0.12	-0.11	0.17
1134	1.31	7.03	0.34	-0.54	-2.72	1.50	-0.63	0.59	-0.15	0.19	-3.20	-2.06	0.19	0.79	-1.70	-0.16
K0325	8.49	3.64	-0.24	0.44	-2.62	0.86	-0.10	2.71	0.77	0.14	1.89	-4.59	0.94	-0.20	-0.60	0.20
2511A	10.85	9.96	0.83	0.12	-0.05	0.32	-1.62	-0.73	0.30	0.91	0.91	1.70	0.65	-0.61	9.01	0.04
CH382	-7.88	-9.36	-0.61	-0.04	3.68	-3.99	-0.95	-3.00	0.14	0.64	-1.57	-0.96	-1.08	-1.10	5.17	0.11
Z3_87	-8.32	-6.54	-3.76	-0.23	-0.90	-4.87	0.78	-5.33	-0.11	-0.89	-1.41	-0.85	-2.45	-0.74	-3.60	-0.28
M60	-10.16	-8.72	-2.62	-0.49	-2.70	-5.53	-0.47	-5.48	-0.61	0.09	-4.06	-0.49	-2.09	-0.52	-2.53	-0.26
S121	-12.86	-12.45	-2.88	-0.28	-2.88	-3.47	0.52	-3.98	-0.21	-0.49	-2.09	-0.63	-0.20	-0.59	-1.59	-0.11
P2237	-5.94	1.43	0.20	0.04	-3.54	-0.86	0.38	-0.04	0.27	0.17	-0.26	-0.30	-0.23	-0.18	-0.48	-0.02
K3841	4.44	-2.22	1.50	0.12	4.61	-0.90	0.66	1.44	0.54	-0.28	-1.23	1.05	0.10	-1.27	0.86	0.08

SD7928	-2.15	-9.70	-2.43	-0.22	-1.62	-3.09	0.17	-1.25	0.27	0.13	-1.90	-2.00	-0.32	-1.16	0.73	-0.05
SD8738	18.88	11.06	-0.84	-0.06	1.43	-1.39	0.46	-0.55	0.42	-0.04	-1.16	-3.17	0.57	1.60	0.38	0.07
K10	16.12	0.92	0.42	-0.04	1.97	-0.71	-1.49	0.41	0.59	0.20	-1.51	-1.15	0.29	0.65	2.56	0.18
Shen3117	14.37	8.80	1.02	0.48	0.56	4.47	0.90	-0.48	0.26	0.06	4.36	0.76	0.80	-0.44	3.16	-0.01
Dan1133	-10.37	-12.79	-1.99	-0.40	-0.67	-5.61	-0.51	-1.25	-1.02	-0.30	-2.94	1.77	-0.81	-0.56	-2.96	-0.33
Dan37	6.15	9.34	0.63	0.36	-0.62	0.46	1.46	0.49	0.39	-0.09	2.14	0.39	2.04	-0.20	-1.09	0.08
M03	-0.27	-7.59	-1.69	-0.26	-1.58	-5.62	-0.41	-1.11	0.15	-0.54	-3.07	-1.78	-1.82	0.19	-2.27	-0.15
M5972	14.30	13.71	-0.68	0.47	-2.22	-1.79	1.15	-0.90	0.78	-0.19	2.53	-1.40	-0.32	-0.25	0.79	0.28
Ming84	-14.77	-4.52	0.46	0.11	0.14	0.34	-0.88	1.53	0.64	0.09	-0.61	-0.81	0.25	-1.27	2.41	0.08
Ming71	-7.01	-5.70	-1.99	0.22	-3.72	-4.45	0.42	-0.76	0.73	0.16	0.35	-2.77	-0.66	-0.65	1.17	0.01
T0278	-4.91	-11.16	-0.42	0.15	-0.89	-2.29	1.42	-0.96	0.56	-0.46	1.16	2.14	0.31	0.54	-0.90	-0.09
TieT0403	7.60	6.77	0.37	0.46	-0.75	2.04	-0.06	0.87	0.72	0.87	2.88	-2.47	0.46	-0.99	5.38	0.37
M53	-2.02	-6.95	-2.55	-0.41	-3.27	-5.11	-0.42	-4.68	-0.23	-0.19	-4.24	-2.86	-1.89	-0.92	-2.60	-0.19
LK11	-7.08	-0.04	2.79	-0.32	2.16	1.94	-1.04	0.92	-0.23	-0.02	-2.72	4.35	1.67	-0.11	3.26	-0.04
S127	22.89	8.47	0.33	0.37	-0.39	-1.61	0.55	0.35	0.52	-0.17	3.80	0.54	0.41	0.01	0.82	0.19
Liao8821	-4.02	-3.70	0.28	-0.24	0.84	0.76	-0.29	0.14	-0.26	0.11	-0.46	-1.16	-0.34	-0.85	0.24	-0.32
Shen391	-7.63	-8.46	-1.31	-0.16	-2.86	-1.76	-0.06	-2.35	0.02	-0.43	-2.00	0.69	-2.46	-0.39	0.74	-0.29
Shen8078	9.94	-1.65	-0.44	0.42	0.88	0.47	1.09	-0.77	0.62	0.30	1.17	-2.16	0.29	-0.34	4.11	0.13
Tie84	14.09	20.10	-0.85	0.14	2.08	3.60	1.24	0.02	-0.32	-0.56	-1.06	-1.01	-1.22	0.66	-4.09	-0.08
Lv9W	11.05	3.98	-0.04	0.56	1.52	4.80	1.87	1.77	1.11	-0.32	2.33	-3.65	0.02	-0.34	-1.41	-0.08
9137	11.53	7.08	0.91	0.26	-3.72	0.61	0.45	1.22	0.44	-0.14	1.27	-0.54	0.18	0.53	1.28	0.06
391	-8.13	-13.19	-0.99	-0.06	5.40	-2.03	-0.25	-1.65	-0.51	0.15	-0.70	-0.54	-0.67	-0.64	0.49	0.01
XKZ101_1	-47.41	-30.35	-3.43	-0.09	-4.98	-2.30	0.90	-2.26	-0.43	-0.14	0.50	-0.66	-1.87	-1.20	-5.34	-0.39
XKZA34	-18.04	-5.19	-0.44	-0.57	0.68	0.76	-0.47	2.12	-0.75	-0.50	-2.09	-1.28	-1.15	0.33	-3.36	-0.12
KL3	-9.66	-4.73	-0.53	-0.24	2.68	0.95	0.42	0.58	-0.43	-0.36	-1.95	-0.46	0.16	-0.60	-3.97	-0.16
XKZ167_1	-40.58	-12.79	-2.65	-0.02	-3.67	0.23	-0.88	-1.68	-0.27	0.50	-0.28	-0.91	-1.09	-0.63	1.84	-0.11
GLMo17	-1.40	-2.87	-0.22	-0.27	0.68	-2.25	-0.48	1.23	-0.80	-0.34	-1.03	-0.11	-1.28	0.25	-1.84	-0.34
JZL610	-5.29	-5.14	0.92	0.12	0.84	-0.49	-0.96	1.46	0.03	0.31	-0.30	0.59	0.34	0.27	2.39	0.02
Chang7_2	4.93	19.49	-0.70	0.44	-2.69	5.75	0.27	-1.59	0.73	0.03	1.90	-3.95	-0.02	0.37	0.45	0.28
KH786	-2.55	-3.23	-2.12	-0.20	-1.46	-4.11	0.43	0.15	-0.15	0.15	-0.60	3.00	-2.38	0.65	1.98	0.05
KH467	13.46	-2.31	1.27	0.26	2.02	-1.92	1.23	1.01	0.69	-0.27	2.34	-0.96	-0.90	-0.67	0.11	0.26
773_2G	-10.14	-1.31	-0.80	-0.03	3.06	0.64	-0.66	0.38	0.03	0.28	-0.51	-3.99	-2.14	-0.43	-0.98	-0.16
Zha461	-0.59	1.22	-0.27	0.07	-0.51	-0.34	-0.12	0.31	-0.21	0.64	1.29	-1.46	-1.19	0.95	3.05	-0.07
917	2.46	0.86	1.87	-0.46	2.48	-1.66	-1.55	1.44	-0.75	0.24	-2.07	0.95	-1.14	0.31	-0.39	-0.12
Hei2	0.90	10.28	0.99	0.14	0.37	1.95	-0.39	3.23	-0.05	0.84	0.95	-2.83	-0.81	0.54	1.99	0.20
KL4	-9.22	-7.24	2.10	-0.51	1.74	-1.41	-1.23	2.69	-0.38	0.44	-4.17	-1.41	1.13	-0.34	0.81	0.03
K454	-11.81	-9.71	-0.15	-0.25	1.42	-3.86	-0.89	-0.55	-0.27	-0.04	-1.50	-2.14	-0.36	-0.15	-0.96	-0.05
M502	-3.89	-12.84	-1.52	-0.33	-0.90	-4.60	-0.41	-2.00	-0.71	-0.43	-2.44	1.78	-3.09	0.27	-2.89	-0.29
7857	-9.93	-7.76	-1.76	-0.25	1.09	0.68	0.64	0.04	-0.32	-0.33	-0.85	0.44	-0.51	0.39	-3.26	-0.07
Xin444	4.02	9.46	0.98	0.19	1.37	0.68	-0.66	0.82	0.12	0.48	0.46	-0.88	-0.15	0.42	2.99	0.28
Zong31	12.87	9.94	2.03	-0.06	3.57	3.06	-0.21	2.13	-0.02	0.14	-0.04	1.51	1.95	0.22	3.31	-0.01
X178	17.63	25.94	0.70	0.06	-5.08	2.64	0.16	-1.60	0.23	-0.21	0.23	-1.08	0.45	1.21	0.05	0.18
HuangC	-5.25	4.42	1.03	-0.27	-0.52	2.69	-0.97	-6.39	-1.24	0.51	-0.67	14.27	0.08	-1.15	9.20	-0.17
P138	10.54	21.04	0.36	0.31	-0.80	2.01	0.85	1.79	0.26	0.79	2.16	-4.24	0.26	-0.09	-0.31	0.12

Zhong451	13.73	6.42	-0.50	0.29	-1.32	-0.39	2.10	3.75	1.29	-1.61	1.20	-4.97	1.41	-0.09	-6.24	0.21
ZZ01	20.95	16.48	3.47	-0.09	0.75	0.13	-0.62	1.69	-0.34	0.08	1.10	2.44	-0.15	1.69	-0.09	0.16
Long53	-12.60	-10.11	-1.18	-0.08	-0.17	-1.00	0.11	-1.10	0.42	-0.22	-0.42	1.09	0.24	-1.05	-0.85	-0.10
HZS	9.42	18.25	-0.19	0.36	-0.58	6.23	0.14	-1.02	-0.02	0.19	2.48	-1.78	-0.42	0.15	0.64	0.16
5213	9.02	-3.65	-0.02	0.09	0.63	-1.72	1.28	-0.05	0.42	-0.07	1.74	0.66	-1.36	-0.21	0.61	-0.04
CN165	14.34	12.42	0.45	0.12	0.68	-0.55	1.45	0.73	-0.05	-0.70	1.94	0.87	0.66	0.40	-2.43	0.07
CN4379	-3.13	12.59	-0.56	0.11	-1.55	2.68	-0.74	0.14	0.42	0.59	0.95	-1.78	0.30	0.27	-0.37	0.10
Qi318	15.12	10.82	2.09	-0.04	0.25	1.51	-0.68	0.52	-0.51	0.34	0.85	2.26	0.78	0.26	1.62	0.05
YFH	0.72	15.02	-1.22	0.17	-1.49	4.22	0.07	1.53	-0.15	0.48	1.20	-3.14	-0.16	0.43	-0.51	0.12
P007	4.55	4.02	-0.17	0.14	-0.62	-1.11	0.27	0.76	0.53	-0.07	1.70	-2.86	0.88	0.12	1.04	0.23
F349	5.84	7.58	1.19	0.19	-1.19	0.30	0.33	0.47	0.52	0.32	1.75	1.49	0.45	0.34	1.25	0.10
P25	5.49	12.61	-0.43	0.08	-2.02	-1.72	-0.76	-1.05	-0.42	0.15	1.93	-1.04	1.27	0.82	1.12	-0.03
Qi205	-6.25	7.11	-1.53	-0.16	-1.30	0.41	0.41	-0.57	-0.13	0.13	-0.48	-0.26	0.01	0.63	-0.80	-0.12
Fu706	1.54	1.02	1.40	-0.29	4.03	2.02	-1.54	1.92	-0.64	0.46	-1.63	-0.41	0.30	0.96	1.17	-0.29
CA375	4.10	9.66	0.89	-0.04	-0.34	4.31	0.49	1.92	0.03	-0.66	-0.14	-1.01	2.77	-0.60	-3.97	0.22
Jing501	1.07	11.51	-0.26	0.18	-2.58	3.64	-0.51	-0.26	-0.12	0.55	1.31	-1.53	-0.62	0.71	0.96	0.07
Jing24	4.88	12.71	0.17	0.06	1.63	2.06	-1.48	-0.56	0.15	0.37	0.45	-0.96	-0.75	0.92	6.26	0.19
Jing89	-4.03	-3.11	0.37	-0.03	-0.32	2.22	0.16	-0.95	0.02	0.31	1.25	0.74	-0.10	-0.41	0.26	0.09
Jing572	-5.50	-1.84	0.05	0.25	-2.95	-1.03	-0.66	-1.27	0.60	0.37	0.06	-0.11	-0.58	-0.31	5.05	0.24
NH60	1.72	-10.12	-2.00	-0.29	-2.63	-3.56	-0.56	-4.10	-0.17	0.03	-2.25	-0.64	-1.18	-0.25	-0.15	-0.20
Jing724	9.71	-2.89	-1.18	-0.08	-0.23	-5.36	1.11	-3.05	0.00	-0.87	-0.05	3.50	-0.21	0.49	-1.81	-0.04
Jing92	7.12	9.90	0.53	0.43	-1.32	2.19	0.31	1.68	0.61	1.03	3.10	-2.21	1.89	-0.89	4.90	0.36
Jing725	-6.12	-12.10	-3.70	-0.30	-1.46	-4.31	0.56	-3.08	0.03	-0.79	-0.48	0.29	-1.12	-0.47	-2.99	-0.27
Aijing525	18.30	17.94	2.30	0.18	0.08	8.71	-0.50	1.51	0.19	0.19	0.04	0.18	1.19	0.77	3.35	0.24
PH6AT	6.44	-3.74	0.53	0.20	0.11	-1.70	1.19	3.05	0.78	-0.14	-0.02	-1.60	1.30	-0.08	-1.47	0.18
He344	-15.86	-14.94	-0.43	-0.19	1.96	-2.60	-1.00	-0.99	-0.42	-0.17	-0.75	-0.74	-0.18	0.19	-0.89	-0.04
C103	2.60	6.57	1.47	-0.07	0.65	3.93	-0.61	2.23	-0.52	0.36	-0.36	-1.91	0.25	-1.41	-0.66	0.06
Zheng58	-22.77	-11.27	0.16	0.17	-1.37	-1.42	-0.59	0.05	0.31	0.81	1.09	0.30	-0.01	-1.17	2.43	-0.08
XL21	-12.56	-1.64	0.16	0.13	-1.29	1.60	0.01	0.82	0.64	-0.50	-0.64	1.33	-0.40	-0.38	0.87	0.07
H2671	0.70	-0.38	0.34	-0.20	0.30	-2.87	-0.82	0.40	-0.03	0.08	-1.25	-2.35	-1.17	0.70	-2.28	-0.09
CL11	15.63	19.55	0.50	-0.01	-0.53	2.00	-0.55	0.97	0.27	0.49	0.28	-0.93	-0.23	-0.12	3.36	0.09
NG5	28.23	20.76	0.85	0.44	-1.62	2.89	1.56	0.90	1.11	-0.16	1.83	1.41	-0.04	0.55	1.71	0.24
HD568	15.32	16.27	-0.30	0.18	-2.84	-0.21	1.10	1.06	0.43	-0.35	0.74	-2.39	0.32	-0.10	-0.67	0.17
11DM124	-7.59	-1.46	-2.09	-0.27	-5.41	-3.84	-0.84	-1.49	-0.42	-0.48	-2.46	-0.49	-2.20	-0.07	-5.72	-0.18
CA616	-17.85	-9.04	-2.48	-0.18	-3.37	-3.19	-0.36	-0.93	0.17	-0.10	-1.54	3.20	-1.97	-0.24	2.50	0.07
Ji6003	0.20	-6.43	0.33	-0.11	1.39	-2.79	-0.08	-1.79	-0.36	0.21	0.88	1.35	1.93	-0.28	-0.72	-0.18
8941	-31.58	-18.13	-2.53	-0.06	1.48	1.09	-0.14	-2.12	-0.32	0.65	-0.17	1.08	0.33	-0.94	-0.94	-0.23
Ming2325	19.97	4.48	-0.36	0.16	-1.26	-0.28	0.26	1.64	1.34	-0.32	1.10	-1.85	2.12	0.48	0.78	-0.22
S5137	25.42	18.34	1.70	0.04	-0.63	3.77	-0.59	1.54	-0.24	0.40	1.42	1.27	0.84	0.36	0.07	-0.18
F2001	2.38	-16.38	-2.84	0.00	-2.40	-2.01	0.81	2.49	-0.53	-0.16	-0.59	-7.36	-0.39	0.19	-3.23	-0.45
20143	19.47	-1.24	2.79	-0.08	5.14	-0.25	0.43	1.95	0.30	-0.36	-1.49	2.94	1.22	-0.60	0.65	0.18
Cheng60	-14.36	-23.77	-3.44	-0.08	-0.55	-2.13	0.30	-0.51	-1.23	-0.29	1.11	4.18	-2.64	-1.21	2.21	-0.06
Cheng53	10.81	9.06	0.53	0.49	2.56	2.48	0.34	2.43	0.34	1.20	3.10	-2.65	0.60	-0.68	4.08	0.25
Chong72	0.54	-2.83	0.25	0.47	3.67	1.20	1.67	2.11	0.30	-1.12	3.78	-1.80	1.12	0.12	-2.52	0.13

NP01200	-9.85	-25.03	0.98	-0.16	0.58	0.33	-0.98	-1.90	-0.90	0.35	-2.12	6.33	0.64	0.71	2.32	-0.16
7884-Ht	-6.76	8.25	2.39	-0.16	4.88	1.52	0.26	5.35	0.67	-0.29	-1.15	-2.48	1.51	0.12	-1.00	0.32
Dong6002	-1.28	3.10	0.04	0.10	1.24	1.00	-0.22	-0.47	0.19	-0.11	-0.75	-1.67	-0.19	0.08	0.34	0.02
H2671	2.52	-4.21	2.06	-0.18	-0.24	-3.27	-0.63	2.70	0.14	-0.06	-1.77	-2.71	-0.84	1.93	4.82	-0.20
Ji846	-10.92	-4.13	-2.72	-0.79	-2.81	-0.68	-0.62	-5.14	-1.24	0.30	-3.04	0.39	0.53	-0.69	-1.22	-0.26
KL2	-12.13	-10.81	-1.85	-0.15	2.46	1.75	0.92	-1.98	-0.65	-1.01	-0.07	-0.19	-0.35	0.85	-5.27	-0.14
KL6	-21.94	-26.97	0.38	-0.51	4.80	-1.17	-1.49	1.75	-1.92	0.35	-2.23	-0.01	-1.82	-0.61	-7.03	-0.18
D3_1	-6.06	-6.08	0.52	-0.05	0.48	-1.45	-0.08	-1.97	-0.05	-0.26	-1.10	4.74	0.35	-1.13	4.01	-0.05
D5_2	4.97	3.97	0.78	0.62	2.45	-0.52	0.13	1.15	0.80	0.38	5.11	-0.21	2.48	0.05	3.45	-0.07
HR30	-23.11	-10.11	1.35	-0.13	1.76	0.93	-1.25	3.14	-0.60	0.09	-2.76	-1.12	0.29	0.67	-3.30	-0.10
KWS10_73	-11.86	-26.61	-0.41	-0.43	3.01	-0.29	-1.28	-1.96	-2.11	0.37	-1.41	2.13	-0.46	0.45	-3.18	-0.37
PHB1M	9.44	-0.86	-0.33	-0.14	-1.26	-2.95	-0.06	-1.09	-0.18	-0.13	-0.13	2.05	0.60	0.12	-0.01	0.00
KWS49	-27.59	-29.54	0.10	-0.42	1.75	1.24	-1.10	-0.80	-1.37	0.15	-3.16	2.36	0.60	0.20	-1.15	-0.10
Jia33	11.18	0.30	1.01	0.06	-1.93	0.34	0.25	1.72	0.31	0.55	-0.26	-0.90	-0.32	0.48	-0.18	0.22
Jia28	4.25	4.16	0.36	0.09	2.08	-2.73	0.34	-0.08	0.79	-0.26	0.84	-0.75	-0.12	0.32	1.62	-0.05
KW5G321	-14.32	-1.25	-1.24	-0.29	-4.09	-3.11	0.03	-0.89	-0.17	0.00	-1.84	-0.07	-1.74	0.63	-2.42	-0.05
KW1A139	-20.26	-25.60	1.41	-0.30	3.77	3.14	-0.49	0.15	-1.22	-0.16	-1.54	4.68	1.23	0.53	-1.88	-0.01
H1208	-17.75	-5.52	-0.58	0.22	1.52	1.81	-0.60	-2.57	0.02	0.79	2.34	1.91	2.64	-0.76	5.43	-0.12
L203	-11.12	-6.45	-0.19	-0.08	-0.39	-1.90	-0.85	-0.52	0.48	-0.34	-1.91	-2.50	0.01	-0.23	0.99	-0.01
81162	-7.48	-3.38	0.71	-0.16	0.25	-1.37	-0.97	-1.81	-0.03	0.59	-1.54	1.37	0.12	-0.54	4.15	-0.03
8638	-15.09	-4.89	-1.52	-0.04	-4.72	1.60	-0.40	0.92	0.15	-0.72	-1.81	-3.71	-0.19	-0.58	-2.86	-0.02
833	-11.35	-8.28	-0.76	-0.05	0.41	1.57	-1.12	1.69	0.13	0.83	-0.57	0.39	-0.13	0.15	3.66	-0.04
ZhongM_8	-1.47	-7.21	1.74	-0.19	3.12	-2.21	-1.35	2.00	-1.02	0.25	0.33	0.41	-0.92	-0.99	-0.88	-0.10
9F592	12.69	12.76	1.15	0.19	0.38	-0.25	-1.02	0.17	0.22	0.31	1.58	0.43	0.82	0.00	3.73	0.15
6F576	-9.20	-10.48	-1.69	-0.14	-2.24	-0.78	-0.77	0.92	0.41	0.02	-2.63	-3.71	-1.38	-0.18	-0.99	0.04
KL613	-16.03	-7.85	0.82	-0.43	0.82	0.46	-0.73	-0.53	-0.36	-0.09	-3.39	-0.93	0.00	0.28	-2.21	-0.11
385_1	-40.59	-29.03	-1.42	0.29	-0.29	-1.72	-0.53	-0.51	-0.64	0.66	1.24	-2.31	0.74	-0.96	-1.42	-0.08
HA25	-28.35	-30.57	-3.01	0.02	1.47	-4.02	-0.79	-4.68	-0.97	0.38	1.63	5.33	-0.49	-0.28	5.03	-0.17
KL45	-25.30	-12.80	-0.40	-0.53	-0.54	-3.61	-0.61	-1.26	-0.54	-0.02	-5.13	-1.26	-1.09	0.61	-4.48	-0.11
Ji63	4.84	7.19	-0.43	-0.04	2.09	2.52	0.49	-3.91	-0.27	-0.43	0.04	1.58	0.39	-0.63	1.62	-0.05
Men14	-16.93	-7.95	-0.76	-0.19	-1.70	1.86	1.10	2.14	-0.38	0.03	-1.70	-2.53	0.92	-0.58	-5.18	-0.21
Xi14	-11.36	-9.78	-0.21	0.01	0.58	-0.78	1.22	2.20	-0.33	-0.86	0.32	-0.71	-0.82	-0.58	-5.26	-0.04
Mo17	14.93	8.05	2.80	-0.22	3.39	-1.31	-0.69	2.48	-0.24	0.26	-2.04	0.41	0.24	0.28	-0.01	0.21
L237	6.45	-0.07	0.35	0.19	0.62	-1.75	0.06	-1.73	0.38	-0.15	0.33	-0.36	0.84	0.73	1.29	0.23
ZaC546	-9.96	-11.33	-2.86	-0.45	1.57	1.86	0.16	-6.64	-1.31	-0.31	-1.48	3.26	0.06	-0.11	-2.78	-0.38
70_104	7.29	6.83	-0.59	0.17	0.58	-2.39	1.53	2.56	0.71	-0.19	0.18	-3.14	0.82	0.58	-2.77	-0.04
L105	4.64	-1.14	1.83	-0.12	2.54	1.65	-0.26	1.74	-1.53	0.11	0.96	4.29	0.09	0.98	-1.72	0.00
Zi330	18.65	8.78	2.35	-0.01	3.60	4.38	0.36	-0.49	-0.12	-0.25	0.29	2.57	1.02	0.46	0.02	0.26
446	1.02	-6.19	0.24	-0.13	1.65	3.73	0.36	-0.92	0.14	-0.66	-1.72	-2.30	0.12	-0.18	-4.75	0.09
Ji853	19.09	7.24	1.18	-0.10	2.96	-0.93	-0.94	-1.03	0.19	0.42	-1.30	1.59	0.36	-0.04	3.40	0.09
434	-11.95	-11.14	-1.97	0.02	0.48	1.79	0.29	-2.87	-0.04	0.08	-0.20	2.47	0.30	-0.84	2.14	-0.12
4F1	4.92	-0.09	0.69	-0.23	2.29	1.31	-0.15	-2.08	-0.89	-0.24	-0.18	3.80	-0.10	0.83	-0.05	-0.15
444	1.91	7.59	0.36	0.08	1.47	0.71	-0.10	0.36	-0.15	0.28	0.03	-0.42	-0.66	0.32	2.24	0.18
Ji002	2.59	11.97	1.40	0.15	-1.20	2.46	0.66	1.65	-0.10	-0.08	1.76	1.59	-0.64	0.86	-1.33	0.18

Ying64	-21.18	-27.54	-1.59	-0.56	-0.10	0.89	-0.93	-0.79	-0.90	0.08	-3.70	0.77	0.07	-1.29	-1.09	-0.28
S8_101	12.86	4.69	0.64	0.12	-0.62	-2.52	-0.28	3.12	0.11	-0.12	1.97	-0.48	1.35	-0.32	-0.87	0.14
7922	4.43	12.62	0.44	-0.16	-4.56	-0.63	-0.01	-0.04	-0.04	-0.11	-1.24	-1.73	-0.76	0.64	-3.27	-0.07
7884_7Ht	-9.91	6.02	1.95	0.04	4.94	0.23	0.44	3.68	0.27	-0.53	0.38	0.06	0.47	0.46	-4.79	0.17
96478	-13.53	-8.33	0.38	-0.19	-2.23	2.78	-1.15	1.23	0.00	0.07	-0.90	-0.64	0.02	-0.60	-2.16	0.09
Ji992	17.19	6.86	1.93	-0.23	2.79	-1.91	-1.21	1.93	-0.26	-0.09	-1.78	2.35	2.07	0.35	0.19	-0.08
Ji1037	19.06	9.32	2.82	-0.20	5.02	-1.52	-0.86	2.63	-0.39	0.47	-1.27	0.63	0.41	0.74	-1.03	0.11
Si_287	-6.60	-6.55	-0.48	-0.03	2.72	-2.24	-0.67	-1.86	0.24	0.12	-0.90	1.23	-0.78	0.08	4.26	0.11
W9706	1.58	-0.34	0.96	-0.24	0.34	-0.75	-1.09	-3.11	0.10	0.60	-2.18	2.25	0.67	-0.46	3.15	-0.18
8902	-13.88	-14.76	-0.57	-0.29	-2.41	-1.45	-0.04	-0.92	-0.63	0.11	-1.32	2.52	-0.09	-0.66	1.35	-0.22
Cheng351	-4.55	-5.18	-0.54	0.07	-0.13	2.13	0.66	1.85	0.25	-0.11	1.29	-0.96	-0.54	0.76	-2.63	0.10
Dan891	-2.96	-6.30	0.70	-0.22	0.17	-1.55	-0.54	-2.37	-0.61	0.25	-0.23	3.29	1.04	-0.04	4.26	-0.32
D22	9.64	11.39	-0.61	0.31	-3.12	0.78	0.38	-1.01	0.67	-0.42	1.32	-3.09	-1.52	0.19	-0.67	0.14
KX	-15.13	-7.29	-1.01	-0.04	-1.79	-1.64	-0.19	-0.42	-0.36	-0.30	0.17	2.33	-0.24	-0.20	-0.72	-0.11
Ji046	10.51	5.92	0.78	0.32	-2.38	-1.04	0.66	0.73	0.09	0.11	3.05	1.66	-0.45	0.18	1.26	0.18
Dan598	21.08	15.90	0.05	0.85	0.21	5.08	1.61	2.38	1.16	0.26	5.60	-1.93	0.40	-0.29	-0.07	0.44
XF	-6.17	-2.16	-3.02	-0.14	-2.69	-0.84	-0.49	-2.26	0.04	-0.35	-1.21	2.21	-0.69	0.39	1.47	-0.15
634150	-1.42	-3.81	-0.97	-0.20	-0.05	-3.26	-1.08	-1.79	0.05	0.02	-1.65	0.59	-0.91	0.20	0.22	-0.29
TM	12.72	10.48	0.73	0.34	0.97	3.86	-0.59	-1.67	-0.13	0.70	2.54	2.16	-0.26	1.37	6.24	0.32
M407	-20.09	-12.82	-3.12	-0.38	-5.43	-3.44	-0.25	-3.65	0.00	-0.33	-3.31	-0.74	-3.27	-0.51	-5.34	-0.28
F62	1.06	-2.58	-0.50	0.44	0.88	1.42	1.17	-0.02	0.76	-0.52	3.06	-0.84	1.81	0.07	-0.78	-0.31
Dan340	5.60	7.19	1.50	0.23	-0.05	2.78	1.12	2.29	0.51	-0.18	4.11	1.14	0.37	-0.38	0.12	0.23
DG11A	-13.00	-0.54	0.71	-0.49	-0.79	2.16	-1.33	0.29	-0.82	0.08	-2.17	-0.44	-0.44	0.99	-2.76	-0.10
Si428	-9.60	-6.85	-1.90	0.13	-1.51	1.61	1.30	-2.35	0.06	-0.11	2.44	1.18	-0.43	-0.60	-1.53	-0.12
Cheng18	-32.20	-24.44	-1.84	-0.53	-2.47	-1.16	-0.36	1.46	-0.69	-0.23	-3.42	-5.02	-2.35	0.90	-7.90	-0.15
Si144	-8.51	-4.75	-0.26	-0.20	-2.25	-1.19	-1.03	1.49	0.03	-0.17	-2.85	-1.43	-0.97	-0.63	-1.35	-0.01
HYM	-1.35	3.86	-1.94	-0.04	-1.51	0.40	-0.67	-0.89	0.42	0.48	-0.19	-4.79	-2.19	0.01	-0.65	-0.12
Lv28	5.95	8.28	-0.66	-0.08	-4.40	3.61	1.07	-0.76	0.56	-0.56	-1.04	-3.17	2.20	0.00	-4.82	0.16
E28	-2.67	3.10	-0.28	0.06	-2.55	2.75	0.18	1.10	0.46	-0.42	-1.19	-2.26	-1.11	0.45	-5.12	0.01
Mo17Ht	6.74	1.98	1.11	-0.30	2.89	-1.34	-0.91	2.89	-0.11	-0.15	-2.03	0.02	0.16	0.27	0.20	0.08
Shen5003	-16.10	-5.04	0.00	0.25	0.04	3.87	0.40	0.93	0.29	-0.10	2.30	-1.27	0.98	-1.02	-1.57	-0.05
KYC8605	10.99	2.62	-0.91	0.32	0.69	-0.67	1.86	1.45	1.10	-0.34	1.29	-0.47	0.75	0.21	-1.56	0.16
7_61	11.01	13.07	0.86	0.31	-0.73	4.33	0.28	0.69	0.33	-0.22	1.81	-0.82	1.21	-0.21	0.12	0.26
Fu746Mu	-5.77	0.39	1.87	-0.16	5.56	0.59	-0.63	-0.95	-0.52	0.08	-1.08	0.88	1.03	1.64	0.21	-0.26
Liao2345	-11.18	-5.48	-1.18	-0.13	-0.51	-0.70	-0.06	-0.89	0.21	-0.13	-1.29	2.98	-0.79	0.01	-0.18	-0.13
Q1261	15.35	11.69	0.22	0.43	1.03	-0.27	-0.40	1.40	-0.14	0.61	4.29	0.73	1.05	0.57	5.86	0.24
Shen137	14.18	19.77	2.53	0.34	1.00	4.32	-0.14	0.76	-0.08	0.45	0.44	0.75	1.21	0.65	1.44	0.23
Dan9046	19.45	12.60	-0.18	0.17	-7.89	1.08	0.07	0.76	0.79	-0.28	-1.40	-0.15	-2.23	1.30	0.06	-0.11
8112	4.71	6.54	-0.32	-0.29	-2.36	2.00	-0.63	1.19	-0.47	-0.11	-1.33	-0.58	0.25	-0.44	-1.84	-0.04
Liao1412	9.93	-2.87	1.14	0.20	0.57	2.04	1.24	-0.57	0.07	-0.61	0.94	-0.66	0.10	0.55	-3.01	0.12
LD175_1	7.29	1.02	0.63	0.00	-1.63	1.08	0.08	-0.01	0.39	0.41	0.62	2.07	2.10	-0.28	2.23	-0.07
LD61	5.82	2.60	-0.20	0.64	-0.23	0.91	2.92	-0.76	1.34	-0.64	4.14	0.60	0.40	-1.17	0.99	-0.18
D26	13.68	8.12	0.40	0.27	-1.79	1.38	0.23	2.40	0.53	0.52	2.55	0.46	-0.11	0.85	0.99	0.17
Dan3130	15.41	14.15	2.11	0.26	0.86	0.53	0.45	1.96	0.02	-0.18	3.07	3.60	0.37	0.42	2.10	0.22

Table S2. SCA data of major maize inbred lines in Northeast China.

Line	Tester	PH	EH	EL	ED	TL	TBN	KRN	KNPR	KL	KW	SD	GT	SDR	SL	HKW	YPP
XF	PH6WC	-4.80	-3.85	2.14	0.17	-0.37	2.07	0.35	1.31	0.23	-0.01	-1.43	-1.47	0.94	-0.04	-0.17	0.23
PH6AT	PH4CV	6.18	1.68	1.75	0.14	0.35	0.66	0.59	1.22	0.26	0.19	0.45	-0.23	0.42	-0.16	0.29	0.20
PHB1M	PH4CV	-18.29	-6.68	-1.94	-0.20	-1.33	1.01	-0.20	-1.60	-0.85	-0.32	-1.81	1.55	-1.32	0.13	-2.99	-0.35
ZhongM_8	PH4CV	2.38	-3.00	0.21	0.11	2.50	-0.01	-0.43	-0.88	-0.03	0.60	0.24	1.75	-0.63	-0.37	3.35	-0.25
L237	PH4CV	-2.13	-0.53	-0.45	0.10	-0.63	0.54	0.11	1.00	0.67	-0.24	0.36	-2.51	0.96	0.48	-0.49	0.06
Ying64	PH4CV	-2.27	-2.01	-0.41	-0.05	0.99	-0.58	0.06	-0.16	-0.10	-0.20	-0.69	-0.43	0.48	-0.28	-1.18	-0.03
Dan891	PH4CV	2.57	3.40	0.84	-0.07	-0.30	0.64	-0.57	2.27	0.09	-0.15	-0.46	-1.74	-0.35	-0.19	0.47	0.01
DG11A	PH4CV	-3.22	-2.45	0.06	0.00	-0.99	-0.81	0.56	0.71	-0.04	0.02	-0.17	-0.53	-1.22	-0.34	-0.08	0.02
Fu746Mu	PH4CV	-0.78	-2.43	-0.07	0.04	0.88	-0.30	0.21	-1.29	0.29	-0.43	0.38	-1.98	0.05	0.01	-0.41	-0.09
Dong46	PH4CV	-1.92	0.43	-0.11	0.05	-0.24	-1.02	-0.32	0.41	0.11	0.19	0.84	-0.26	0.38	0.18	0.81	0.11
Dong237	PH4CV	-1.52	-3.80	0.48	-0.02	1.05	0.27	-0.24	-1.31	0.24	-0.65	-0.32	0.18	-0.25	-0.23	1.37	0.02
Hai268	PH4CV	-4.93	1.51	-0.46	0.23	-0.92	0.32	-0.74	0.47	-0.16	0.84	0.27	-0.41	-0.13	0.09	-0.24	-0.11
1134	PH4CV	10.97	1.28	-1.09	0.11	-1.06	-2.24	0.26	-1.35	0.34	-0.67	-0.77	-1.96	0.20	0.75	0.30	-0.04
K10	PH4CV	2.66	-0.16	0.00	0.04	1.21	-0.49	0.33	0.38	-0.06	0.10	0.30	0.38	-0.09	0.33	-0.03	0.07
LK11	PH4CV	-7.36	-3.88	-0.69	0.04	-2.06	-0.28	0.53	-1.68	-0.02	-0.43	0.38	-0.11	0.47	0.27	-0.66	-0.15
KL3	PH4CV	2.81	2.59	-0.47	0.10	1.38	1.42	0.46	0.75	0.26	0.15	-0.02	-1.05	1.32	-0.18	0.33	0.02
KL4	PH4CV	-0.12	-4.02	0.87	-0.04	-1.24	-0.10	0.38	1.09	0.04	-0.39	0.34	-0.76	-0.11	-0.49	0.63	0.12
Long53	PH4CV	-27.47	-13.59	0.51	0.08	3.69	-2.18	2.18	-1.17	0.10	-0.31	1.74	-0.09	0.68	-2.15	-2.88	-0.39
Fu706	PH4CV	-7.15	-1.54	0.09	0.06	1.21	-1.82	-0.19	0.17	0.12	0.07	0.02	-2.12	-0.68	0.52	1.27	-0.03
He344	PH4CV	2.01	4.97	-0.73	0.00	0.52	-0.35	0.21	0.49	0.14	-0.19	-0.86	-1.60	-0.79	-0.10	-1.02	0.03
8941	PH4CV	-0.38	-2.51	-0.69	-0.02	-0.56	-0.15	0.16	0.05	-0.09	-0.16	-0.41	-0.88	-0.06	-0.14	-0.97	0.06
Dong6002	PH4CV	-3.98	-7.34	-0.02	-0.09	0.53	-0.37	0.03	0.56	-0.40	-0.38	-0.99	-1.25	0.63	0.47	-3.37	-0.11
Ji846	PH4CV	-20.73	-15.08	-5.28	-1.22	0.75	-0.06	0.02	-0.30	-1.20	0.79	-2.07	-3.87	1.67	0.38	-2.30	-0.39
KL2	PH4CV	1.77	1.77	0.48	0.05	0.25	0.02	0.49	2.08	-0.07	0.31	0.05	0.23	0.34	0.63	1.23	0.12
KL6	PH4CV	0.61	-4.03	1.10	0.11	3.73	-0.10	-0.12	2.57	-0.04	-0.11	0.13	-0.38	0.77	0.36	0.38	0.13
D3_1	PH4CV	-9.88	1.81	-1.38	0.00	-1.36	-0.02	-0.34	0.33	-0.06	0.36	0.55	-1.05	-0.30	-0.47	-0.29	-0.11
D5_2	PH4CV	3.79	-4.45	1.97	-0.07	0.59	0.73	0.20	2.25	0.04	-0.24	-0.41	0.05	0.65	-0.59	0.53	-0.11
HR30	PH4CV	-2.56	-2.42	-0.29	0.15	0.68	0.36	0.20	-1.44	-0.11	-0.25	-0.56	-0.43	0.07	-0.43	1.71	-0.04
KWS10_73	PH4CV	-3.23	-4.49	0.22	0.01	1.03	0.20	0.02	0.29	-0.11	-0.05	0.06	-0.47	0.05	-0.01	-1.74	-0.01
KWS49	PH4CV	-2.12	-4.63	0.66	-0.10	-0.04	0.22	0.19	1.01	-0.06	-0.05	0.59	-0.39	0.23	-0.24	-0.14	-0.02
Jia33	PH4CV	8.50	2.13	0.77	0.06	0.26	-0.70	0.09	0.56	0.02	-0.15	0.40	0.41	0.16	0.40	0.49	0.09
Jia28	PH4CV	-2.12	-1.10	-0.04	0.14	-0.26	0.67	0.07	0.32	0.09	0.20	1.05	0.10	-0.32	0.62	1.46	-0.09
KW5G321	PH4CV	-17.68	-4.62	-1.93	-0.22	-2.37	0.65	-0.09	-0.59	-0.05	-0.24	-0.17	-0.35	-1.55	0.16	-0.93	-0.18
KW1A139	PH4CV	1.08	-4.97	-0.18	-0.03	1.30	0.35	-0.19	-0.39	-0.14	0.14	0.02	1.27	0.53	0.12	0.96	-0.02
H1208	PH4CV	3.66	-7.39	-0.91	0.05	1.84	-1.78	0.12	-0.20	-0.02	-0.10	1.05	0.21	0.15	-0.79	-0.40	0.03
L203	PH4CV	2.15	-1.51	-0.20	-0.04	0.21	0.30	0.03	0.39	-0.21	-0.22	-0.11	-1.13	0.59	-0.14	-0.99	0.14
81162	PH4CV	-2.41	0.49	-1.05	0.03	-1.19	-0.23	-0.03	-0.51	0.04	0.07	0.62	-0.54	0.57	-0.23	-0.77	-0.07
8638	PH4CV	-3.53	-4.44	-1.38	-0.03	0.08	-0.38	0.25	-1.35	0.25	0.05	0.34	-1.03	-0.38	-0.02	-0.30	0.01
833	PH4CV	1.27	-2.47	0.08	-0.04	1.28	0.24	-0.36	-0.16	0.06	-0.07	0.74	0.26	0.16	-0.24	0.54	-0.04
9F592	PH4CV	0.10	4.13	0.12	0.06	1.14	0.15	0.17	-1.03	0.31	0.02	-0.15	0.11	0.24	0.50	0.85	0.10
6F576	PH4CV	-3.85	-4.43	-0.93	-0.01	-0.40	0.58	0.12	-0.10	-0.22	-0.03	-0.33	-0.54	-0.54	0.40	-0.91	-0.03
KL613	PH4CV	-1.43	-0.29	0.16	0.11	-0.25	-0.44	0.23	-0.89	0.56	-0.04	0.94	0.47	0.65	0.08	1.57	0.13

385_1	PH4CV	1.01	3.51	0.34	-0.09	0.37	0.17	-0.43	-1.42	-0.20	-0.17	-0.06	1.35	0.23	-0.15	-0.83	0.05
HA25	PH4CV	-3.14	-1.66	-1.21	0.01	0.18	0.71	0.38	-0.07	0.18	0.02	0.05	-0.98	0.27	0.21	-1.09	0.00
KL45	PH4CV	1.77	3.63	-0.71	0.06	-2.00	1.03	-0.12	-0.74	0.29	-0.12	0.17	-0.50	0.38	0.51	-0.11	0.06
Ji63	PH4CV	0.04	-1.83	0.41	0.08	0.52	-1.52	0.21	1.16	-0.32	-0.09	1.00	0.80	-0.70	0.10	-0.34	0.02
Men14	PH4CV	7.85	3.37	1.15	0.09	-0.47	0.91	-0.19	2.05	0.16	-0.25	-0.26	0.23	0.10	-0.12	-0.07	0.04
Xi14	PH4CV	-0.87	0.21	0.14	-0.03	-1.18	0.40	-0.36	0.56	-0.07	0.07	-0.88	0.36	-0.19	0.05	0.28	-0.09
Mo17	PH4CV	0.36	4.32	-0.53	0.08	-0.68	0.45	-0.01	0.55	0.25	-0.05	0.18	-0.34	0.00	-0.02	0.94	-0.01
ZaC546	PH4CV	0.69	1.05	-0.36	0.10	0.87	-0.27	-0.18	0.70	0.18	0.17	0.89	0.30	0.14	-0.24	1.18	-0.03
70_104	PH4CV	3.27	3.79	1.12	-0.04	-0.22	0.48	0.03	1.22	-0.27	-0.32	0.74	-0.14	0.34	0.73	-1.97	-0.13
L105	PH4CV	-3.64	-3.78	-0.96	0.12	0.10	-0.43	-0.06	-1.32	0.03	0.20	1.47	-0.98	-0.07	-0.15	0.33	0.06
Zi330	PH4CV	-1.59	-1.26	0.15	0.07	-0.60	-0.11	0.85	0.07	0.09	0.06	0.16	-1.11	-0.29	-0.33	0.20	0.07
446	PH4CV	2.84	3.55	-0.40	0.05	-0.62	-0.27	0.51	-2.00	0.00	0.09	0.19	-0.12	0.72	-0.26	0.08	0.07
Ji853	PH4CV	-2.20	-3.28	-0.60	-0.01	0.80	-2.07	-0.12	0.11	0.14	-0.08	-0.13	-1.27	-0.81	0.08	-1.06	0.04
434	PH4CV	-6.88	-5.07	-1.67	-0.10	-0.48	0.70	0.05	0.00	-0.06	-0.19	0.68	0.72	-0.48	-0.03	-1.26	0.00
4F1	PH4CV	4.26	4.09	-0.05	-0.02	-1.05	-0.13	0.02	-0.76	0.13	0.37	0.24	-1.75	-0.89	0.69	1.38	-0.12
444	PH4CV	0.58	-1.67	-0.01	0.01	0.11	0.38	0.19	0.36	-0.03	0.04	0.07	-0.07	0.29	-0.05	-0.78	0.04
Ji002	PH4CV	4.26	2.93	1.11	-0.02	-1.20	-0.09	-0.07	0.72	0.09	-0.04	-0.05	0.98	0.26	-0.04	-0.11	0.15
S8_101	PH4CV	1.11	3.98	0.69	-0.09	-0.40	1.38	-0.30	1.30	0.09	0.00	-1.66	0.58	0.23	0.68	-0.07	0.08
7922	PH4CV	-10.10	-3.71	-0.92	-0.17	-1.20	0.62	-0.28	1.30	-0.46	-0.43	-0.64	0.40	0.05	-0.28	-1.28	-0.16
7884_7Ht	PH4CV	-4.93	-0.13	0.22	-0.05	-0.53	0.22	0.07	1.65	-0.12	-0.11	-0.25	-1.48	-0.48	-0.84	-3.79	0.02
96478	PH4CV	-2.97	-2.55	0.12	-0.08	-1.03	0.67	-0.36	-0.09	-0.04	0.07	-0.47	-0.38	-0.45	-0.14	-1.21	0.00
Ji992	PH4CV	-0.90	1.62	-0.45	0.04	0.72	0.20	-0.02	-1.15	0.17	-0.14	0.32	1.44	-0.52	-0.12	0.57	-0.05
Ji1037	PH4CV	1.71	2.95	-0.27	0.04	-0.19	-0.57	0.16	2.32	0.27	-0.23	0.10	-1.49	-0.43	0.07	-1.27	-0.03
Si_287	PH4CV	-5.87	0.50	-0.46	0.00	-0.61	-0.17	-0.44	0.28	0.14	-0.30	-0.11	-0.77	1.00	0.08	0.30	-0.03
W9706	PH4CV	4.10	-3.88	-0.42	0.06	-0.22	-1.13	0.37	0.78	0.38	0.28	0.79	-0.47	0.36	-0.81	2.54	-0.08
8902	PH4CV	-0.54	-4.54	-0.07	-0.11	-0.81	0.71	-0.52	-0.47	-0.50	0.16	0.63	0.98	-0.11	-0.24	-1.22	0.06
Cheng351	PH4CV	-2.70	-0.53	0.21	-0.19	-0.43	0.76	0.21	0.47	-0.07	0.29	-1.41	2.74	0.14	-0.13	-0.01	-0.22
D22	PH4CV	-0.31	-0.87	-1.00	0.07	0.14	0.65	0.06	-0.40	0.21	0.01	1.16	0.48	0.14	0.13	0.82	-0.02
KX	PH4CV	-6.30	0.90	-0.89	0.01	-1.21	0.74	0.50	-1.77	-0.50	-0.64	0.48	-0.69	-0.25	0.11	-2.14	0.04
Ji046	PH4CV	12.52	4.44	0.06	0.05	-0.93	1.35	0.12	0.41	-0.30	0.26	0.59	1.09	0.31	0.51	0.59	-0.02
Dan598	PH4CV	-3.01	-2.43	-0.04	0.01	-0.29	-0.43	0.08	0.76	0.07	0.14	0.10	-0.17	-0.47	-0.14	0.39	0.02
XF	PH4CV	2.54	2.39	-1.38	-0.10	0.13	-0.98	-0.16	0.01	-0.11	0.00	0.84	-0.17	-0.64	-0.07	-0.76	-0.13
634150	PH4CV	0.67	1.72	1.89	0.07	1.29	2.81	0.14	2.47	-0.10	0.37	1.52	-0.28	1.45	0.18	1.69	0.05
TM	PH4CV	-7.48	-3.78	0.30	0.03	-0.44	-1.78	-0.25	-0.04	-0.11	-0.02	0.48	0.86	0.00	0.29	0.73	0.07
M407	PH4CV	16.01	3.57	1.06	0.17	1.13	1.14	-0.01	0.73	0.50	1.09	0.54	-0.50	0.98	0.78	-1.66	0.01
F62	PH4CV	-6.56	-4.10	0.71	0.03	-0.39	-0.49	-0.31	1.25	0.08	-0.06	-0.91	-1.24	0.74	-0.34	-0.75	-0.13
Dan340	PH4CV	3.74	3.12	-0.32	-0.02	-0.33	-0.84	0.76	1.02	0.34	0.22	1.24	-0.61	-0.21	-0.07	-0.81	-0.05
Si428	PH4CV	4.04	5.55	0.24	0.14	0.09	-0.57	-0.22	2.30	-0.47	-0.59	1.39	-0.46	-0.98	0.37	-0.88	0.09
Cheng18	PH4CV	-2.67	-0.14	-1.21	-0.04	-0.22	-0.45	0.10	-0.99	0.00	0.30	0.18	-0.57	0.78	-0.42	-0.71	-0.03
Si144	PH4CV	-5.11	-3.90	-0.14	0.05	1.04	0.13	0.50	-1.21	0.40	-0.17	0.59	0.72	0.17	0.23	0.80	-0.01
HYM	PH4CV	-1.24	-0.55	0.49	0.13	1.64	1.98	0.83	0.43	0.13	0.92	1.07	0.32	1.14	-0.98	-0.35	-0.02
Lv28	PH4CV	7.51	5.00	0.26	-0.06	-0.55	-0.83	-0.18	1.57	-0.02	-0.50	-0.29	0.40	0.05	0.48	0.88	0.02
E28	PH4CV	-0.80	4.06	0.18	0.11	-0.18	0.84	0.24	-0.11	0.24	0.65	1.58	-0.97	-0.40	-0.02	0.04	0.15
Mo17Ht	PH4CV	-1.43	2.92	-0.62	-0.05	-0.61	-0.91	-0.28	0.69	0.30	0.83	-0.32	-2.08	0.16	0.43	0.81	0.03

Shen5003	PH4CV	4.00	7.20	0.43	-0.11	-1.57	-0.34	0.04	1.42	0.07	0.09	-0.12	0.86	-0.53	-0.25	-1.14	-0.14
KYC8605	PH4CV	-8.98	-4.12	0.55	-0.04	0.18	-0.71	-0.22	-1.25	-0.18	-0.42	-0.34	0.83	-0.76	-0.36	-1.03	0.01
7_61	PH4CV	-0.94	0.94	0.00	-0.02	-0.99	-0.87	-0.17	-0.72	-0.28	0.17	0.35	0.88	0.08	0.43	0.88	0.00
Liao2345	PH4CV	-5.42	2.40	-0.48	-0.14	-1.41	0.19	0.35	-1.55	-0.53	-0.87	-0.64	0.97	-0.87	-0.61	-2.95	-0.17
Q1261	PH4CV	-4.27	-4.35	-0.09	-0.01	-0.32	0.96	-0.01	0.17	0.05	0.15	0.63	1.26	0.33	-0.71	1.19	-0.02
Shen137	PH4CV	-0.69	-1.45	-1.03	-0.18	-1.65	1.28	0.29	0.26	-0.08	-0.44	-0.07	-0.49	-0.48	0.10	-1.24	0.00
Dan9046	PH4CV	-8.15	6.28	0.81	-0.03	0.14	-2.13	0.93	-0.06	0.11	0.15	0.03	-0.42	-0.01	0.17	-0.60	-0.29
8112	PH4CV	4.91	1.88	-0.15	-0.18	0.37	0.98	-0.45	0.40	-0.34	0.17	-0.36	-0.11	-0.88	0.07	-2.26	-0.12
Liao1412	PH4CV	0.17	2.41	0.86	0.06	0.03	-0.35	-0.28	1.96	-0.33	-0.19	-0.49	1.45	-0.11	0.67	0.01	0.11
LD175_1	PH4CV	3.65	0.63	-1.83	0.05	-0.14	0.76	0.52	-0.40	-0.01	0.27	0.39	-0.91	0.01	-0.18	-2.76	-0.02
LD61	PH4CV	-7.15	-3.98	-0.26	0.01	-0.44	0.67	-0.05	0.04	0.12	0.11	-0.38	-0.46	0.16	-0.36	-0.49	-0.11
D26	PH4CV	3.03	-1.90	0.87	-0.13	-0.80	1.88	-0.38	0.85	-0.64	0.21	-0.43	4.07	0.45	-0.83	-1.04	0.02
Dan3130	PH4CV	6.48	1.78	1.04	0.15	0.19	-0.31	-0.09	0.52	0.30	0.14	0.57	-2.26	-0.05	-0.29	0.53	0.14
A801	PH4CV	3.23	5.55	-0.02	0.00	-0.95	1.45	0.33	1.89	-0.08	-0.09	0.18	0.71	0.07	0.71	-0.95	-0.09
Liao68	PH4CV	5.46	7.30	-0.13	-0.15	-0.61	0.84	0.28	0.19	0.02	-0.26	-1.01	-0.69	-0.06	0.06	-2.82	-0.03
LIao3180	PH4CV	-8.21	-2.55	-0.72	-0.18	-0.79	1.27	0.17	0.48	-0.36	-0.22	-0.60	0.33	-0.24	-0.23	-2.21	-0.17
K12	PH4CV	-6.46	-11.53	1.73	-0.09	0.48	2.33	-0.25	0.22	-0.15	0.18	0.57	4.69	0.26	-0.59	2.57	-0.33
Shen151	PH4CV	-4.93	-5.18	-0.35	-0.12	-0.34	-1.01	-0.53	1.34	-0.02	0.17	-0.23	-1.69	-0.33	-0.55	-1.55	-0.26
Dan717	PH4CV	-1.99	-6.32	-0.76	0.02	-1.30	0.80	-0.01	1.45	0.23	0.02	0.43	0.48	-0.18	-0.37	-0.69	-0.06
Liao8478	PH4CV	-0.76	0.31	0.22	-0.12	-2.16	0.08	0.06	1.94	-0.13	-0.19	-0.59	1.00	-1.18	-0.07	-1.32	0.01
Zheng22	PH4CV	0.40	-0.04	0.20	0.18	0.92	-0.84	0.33	0.77	0.54	0.15	0.35	-0.99	-0.18	0.48	3.58	0.20
Zhong106	PH4CV	-1.92	0.33	0.22	0.13	-0.62	-0.21	0.11	0.14	0.12	0.12	2.19	0.90	-0.16	0.46	1.28	0.10
Liao3162	PH4CV	0.96	-0.58	-0.31	-0.09	-0.46	0.33	-0.15	-1.76	-0.06	0.01	0.11	-1.03	-0.33	-0.16	-2.32	-0.13
PH09B	PH4CV	-18.83	-6.55	-4.19	-0.08	-3.63	0.53	-0.08	3.07	-0.34	-0.63	-1.13	1.03	-2.39	-0.61	-4.25	-0.16
PHPMO	PH4CV	27.24	11.84	3.37	0.50	3.21	1.15	-0.18	7.21	1.63	0.10	2.45	-1.44	0.54	0.87	4.16	0.39
Dan988	PH4CV	2.86	6.50	0.58	-0.03	0.36	0.34	-0.06	-0.20	-0.20	-0.15	0.72	2.33	-0.70	0.23	-0.55	0.01
DanT138	PH4CV	-1.68	-1.11	0.06	0.07	0.34	1.06	-0.07	0.55	0.04	0.16	0.79	-0.66	-0.78	0.26	-1.67	0.04
Liao6082	PH4CV	2.61	3.33	0.54	-0.14	0.24	0.70	-0.55	0.93	-0.23	0.00	1.01	0.24	-0.42	-0.20	0.16	-0.06
63792	PH4CV	-2.03	-3.51	-1.87	-0.16	-0.05	0.94	0.08	-2.36	-0.40	0.03	1.21	0.72	-1.34	-0.07	-1.69	-0.16
7017	PH4CV	-5.88	1.56	0.75	0.18	-0.42	0.43	0.26	-0.46	0.55	-0.10	1.56	1.46	-0.11	-0.54	3.06	0.22
Dan99C	PH4CV	-3.93	-3.92	0.13	0.03	-0.30	-0.51	-0.40	-0.55	0.15	-0.01	-0.42	-0.23	-0.47	-0.15	-0.65	0.03
Shen3336	PH4CV	1.14	-0.17	0.28	0.16	-0.80	0.58	0.38	1.35	0.33	0.13	0.38	-0.56	-0.68	-0.06	-0.65	0.21
Shen3265	PH4CV	1.64	-3.13	1.60	0.01	-0.34	-0.10	0.30	1.05	-0.34	0.04	1.12	2.42	0.06	0.06	-0.79	0.16
T98103_1	PH4CV	-6.53	-5.56	0.40	-0.11	-0.04	1.15	-0.13	0.05	-0.20	-0.02	-0.24	2.18	0.27	-1.49	0.29	-0.14
Dan360	PH4CV	-0.49	-0.95	-0.72	-0.04	0.49	-2.14	-0.43	0.26	0.03	0.22	-0.47	-0.19	-0.62	-0.08	-0.12	-0.11
CL143	PH4CV	3.16	0.17	0.93	0.03	-0.76	0.30	-0.37	1.63	0.44	-0.15	0.26	0.76	0.29	-0.24	0.81	0.11
CH382	PH4CV	-5.89	-3.95	2.34	-0.11	1.35	-0.23	0.42	4.07	0.36	0.09	0.19	3.02	0.84	-0.66	1.36	-0.11
PH6JM	PH4CV	-10.69	-4.62	-1.91	-0.18	0.10	-0.99	0.35	-1.22	-0.75	-0.29	0.66	1.19	-0.36	-0.69	-4.50	-0.28
Dan6263	PH4CV	0.17	-0.98	0.70	-0.05	-0.86	0.30	-0.43	0.89	0.11	0.18	-0.61	0.70	-0.38	0.20	1.68	0.05
C260	PH4CV	1.95	-2.65	1.46	-0.07	-1.11	1.33	-0.35	0.18	-0.23	0.91	-0.62	-1.23	0.46	-0.15	0.93	-0.23
C168	PH4CV	-1.24	-1.36	0.66	0.09	0.93	0.02	0.36	2.02	0.39	-0.02	0.50	-0.60	0.42	-0.24	-0.04	0.20
Liao7980	PH4CV	4.60	0.24	0.69	-0.12	-0.37	0.88	-0.49	-0.46	-0.03	0.30	-0.74	0.97	1.09	0.83	-0.06	0.01
F12	PH4CV	-4.29	-3.60	-0.09	-0.08	0.58	-0.35	-0.39	-0.52	-0.18	0.34	-0.58	0.46	-0.79	0.07	1.11	-0.13
K0325	PH4CV	1.89	1.22	1.01	-0.07	0.45	-0.48	-0.20	2.83	-0.02	0.12	-0.30	-0.27	0.63	-0.26	0.26	-0.06

2511A	PH4CV	-1.83	0.81	0.22	0.06	1.30	-0.10	0.30	-1.70	0.29	-0.17	0.11	0.69	0.65	-0.02	0.19	0.25
CH382	PH4CV	-2.68	-2.49	-0.50	-0.10	-1.26	0.35	-0.11	-0.81	-0.36	-0.17	-0.25	-0.28	-0.68	-0.06	-1.72	-0.18
Z3_87	PH4CV	8.97	4.82	1.55	0.14	1.20	0.87	0.43	2.01	0.12	0.10	0.37	0.63	0.33	0.30	1.58	0.05
M60	PH4CV	19.82	2.30	0.76	0.04	0.28	0.34	0.13	2.64	0.11	0.03	0.23	0.22	-0.22	0.30	0.62	0.03
S121	PH4CV	29.72	14.11	3.18	0.44	3.12	1.45	0.19	4.52	1.50	-0.08	0.49	-3.50	2.00	0.51	4.21	0.56
P2237	PH4CV	1.73	4.21	-0.46	-0.15	-1.04	-0.50	-0.12	1.04	-0.12	0.04	-0.69	1.06	-0.20	0.04	-1.08	-0.13
K3841	PH4CV	-4.72	-8.80	-0.62	-0.01	-0.10	0.37	0.21	-0.73	-0.01	-0.18	-0.99	-0.47	0.45	-0.23	-0.25	-0.22
SD7928	PH4CV	-16.60	-10.47	-4.79	-0.41	2.01	2.57	-0.27	-0.84	-0.33	0.11	-1.49	-0.01	-2.49	-2.69	-4.03	-0.28
SD8738	PH4CV	8.08	0.22	0.22	0.08	1.69	1.64	0.83	1.49	0.57	0.50	0.82	-0.73	0.54	0.08	1.85	0.06
Shen3117	PH4CV	1.73	2.96	0.86	0.05	-0.88	0.48	0.11	-0.20	0.14	-0.41	0.04	0.23	0.47	0.44	-1.20	0.18
Dan1133	PH4CV	5.51	3.91	-0.34	-0.17	-0.12	0.85	-0.40	-1.01	-0.36	0.26	-0.33	0.75	-0.15	0.69	0.08	-0.02
Dan37	PH4CV	-0.60	1.09	-0.16	-0.04	0.28	-1.75	0.30	-0.81	-0.30	-0.13	0.04	1.11	0.16	-0.27	-0.42	0.05
M03	PH4CV	6.63	4.30	0.44	-0.05	-0.53	0.43	0.07	1.15	-0.47	0.47	0.15	-0.86	0.05	-0.03	-0.14	-0.13
M5972	PH4CV	3.43	5.63	1.24	-0.09	1.03	-0.01	0.03	2.03	-0.14	-0.39	-1.17	0.57	0.57	0.26	0.56	0.16
Ming84	PH4CV	4.63	-0.05	0.16	0.00	0.27	-0.57	0.30	-0.59	-0.16	0.10	0.12	0.79	-0.59	-0.44	-1.52	-0.17
Ming71	PH4CV	-0.38	-0.12	-0.07	-0.10	-1.01	1.14	0.34	-0.89	-0.10	-0.41	-0.87	0.65	0.50	-0.06	0.17	-0.16
T0278	PH4CV	1.96	4.35	0.07	-0.05	-0.10	0.18	-0.09	-1.89	0.06	0.41	-1.29	1.41	0.00	0.53	-0.31	-0.02
TieT0403	PH4CV	-0.51	-0.52	-1.01	0.04	0.72	-1.95	0.08	-1.25	-0.18	-0.34	0.68	-0.16	-0.25	0.33	0.47	-0.03
M53	PH4CV	13.07	1.75	1.04	0.15	2.83	1.04	0.80	4.25	0.41	0.45	0.32	-1.42	0.44	0.15	1.12	0.10
S127	PH4CV	3.82	4.21	-0.13	-0.12	-0.44	0.06	0.47	-2.24	0.01	-0.32	-1.03	0.26	-1.09	0.74	0.52	-0.09
Liao8821	PH4CV	13.90	19.46	-1.24	-0.32	3.22	1.47	0.35	-7.24	-0.82	-0.15	-0.79	2.24	0.17	-0.41	-1.91	-0.30
Shen391	PH4CV	3.73	-1.92	0.84	0.05	-0.36	0.94	0.33	1.19	0.10	0.01	1.21	0.41	-0.21	-0.16	-0.82	0.08
Shen8078	PH4CV	3.47	3.71	0.31	0.02	-0.74	-0.99	-0.18	2.07	0.24	0.64	0.57	1.06	-0.47	-0.32	0.29	0.09
Tie84	PH4CV	3.24	1.30	-0.02	0.47	0.20	0.71	-0.08	1.72	0.09	-0.22	0.93	0.75	-0.53	0.11	2.14	0.05
Lv9W	PH4CV	-5.73	-5.57	0.34	0.05	0.40	-0.46	-0.20	0.73	0.30	0.71	0.62	-1.39	0.88	-0.08	0.95	-0.19
9137	PH4CV	1.20	-4.99	0.23	-0.06	1.17	-0.68	-0.01	0.55	-0.09	-0.07	0.28	0.20	0.31	-0.38	-0.95	-0.13
391	PH4CV	-2.04	2.17	0.58	-0.08	-0.51	-0.29	-0.86	-0.17	0.01	0.53	-1.41	-0.38	-0.05	-0.04	1.26	-0.08
XKZ101_1	PH4CV	-1.96	-1.30	-1.66	-0.02	-0.52	-0.39	0.83	-5.01	-0.28	-0.25	0.36	1.12	-0.06	-0.01	2.31	0.05
XKZA34	PH4CV	-1.38	-0.64	-0.56	0.03	1.01	0.42	0.10	-0.11	0.34	0.39	0.33	0.64	-0.43	0.06	1.21	0.09
XKZ167_1	PH4CV	2.87	-4.29	0.33	0.03	-0.40	-0.01	0.80	-0.76	-0.24	-0.39	0.49	2.00	-0.07	-0.28	0.70	0.06
GLMo17	PH4CV	-0.01	2.58	0.45	0.15	1.02	0.01	0.12	-1.86	0.37	0.47	-0.43	0.29	0.71	0.26	2.95	0.09
JZL610	PH4CV	-3.07	-3.41	-0.04	0.15	0.57	-0.33	-0.28	-0.64	-0.12	-0.18	-0.12	1.16	-0.48	-0.19	0.14	-0.17
Chang7_2	PH4CV	2.33	-2.44	-0.02	0.07	0.35	-2.24	-0.11	-2.00	-0.46	0.47	0.74	-0.55	0.21	-0.07	1.11	0.11
KH786	PH4CV	-20.33	-4.58	-2.88	-0.22	-3.60	1.66	-0.04	-3.43	-0.89	-0.30	-0.90	-0.28	-2.10	-0.90	-3.21	-0.36
KH467	PH4CV	6.51	2.61	0.41	0.03	0.54	0.19	0.51	-1.09	-0.33	0.57	-0.37	-0.66	-0.61	0.12	-0.74	0.13
773_2G	PH4CV	6.31	1.12	-0.57	0.03	-2.08	-1.44	0.02	-3.51	-0.07	-0.48	-0.04	-0.83	-0.41	-0.25	0.24	-0.01
Zha461	PH4CV	-1.42	-0.44	-0.29	0.00	-0.06	1.25	-0.10	0.88	0.01	0.03	0.19	-1.93	0.22	-0.25	-1.79	0.02
917	PH4CV	-0.58	3.06	-0.29	0.15	0.92	-0.32	0.62	-2.96	0.30	-0.12	-0.12	-2.70	0.16	-0.40	0.46	0.01
Hei2	PH4CV	-5.48	-2.07	0.40	0.08	-0.68	0.09	0.26	-1.08	0.22	0.59	-1.62	-8.34	0.22	-0.06	2.07	0.00
K454	PH4CV	-2.61	3.09	-0.64	0.11	0.39	0.55	0.10	-0.68	0.26	-0.13	-0.75	-0.76	-1.24	-0.07	0.93	0.06
M502	PH4CV	0.46	0.64	0.00	0.00	0.59	1.04	-0.16	1.90	-0.01	0.05	0.06	-1.26	0.25	-0.11	-1.09	0.04
7857	PH4CV	6.00	5.16	-0.11	0.04	-1.24	-1.39	-0.57	-0.68	0.29	-0.21	-0.65	2.20	-0.16	0.13	2.62	-0.04
Xin444	PH4CV	-1.67	-3.92	0.23	0.06	0.16	-0.66	0.31	-0.46	-0.26	0.52	-0.10	0.20	0.56	-0.60	0.56	0.06
Zong31	PH4CV	-4.44	-3.42	0.33	0.06	1.21	1.29	0.45	-0.44	-0.01	-0.42	0.18	1.09	0.18	-0.56	0.19	0.06

X178	PH4CV	2.96	1.05	0.23	-0.10	-0.63	0.18	-0.13	-0.77	-0.27	0.56	-1.05	0.45	-0.20	-0.85	0.27	0.04
HuangC	PH4CV	-0.99	0.43	-0.20	0.01	-0.03	0.39	0.06	1.40	0.12	0.02	0.47	-1.92	-0.13	0.01	-1.50	0.00
P138	PH4CV	-0.09	-4.73	0.45	-0.08	1.54	-0.26	-0.06	0.27	0.13	0.39	-1.31	-1.19	0.96	-0.12	-1.04	-0.17
Zhong451	PH4CV	6.98	0.15	0.52	-0.20	0.10	-1.07	-0.95	1.25	-0.24	0.11	-1.09	-0.05	0.56	0.15	-0.13	-0.09
ZZ01	PH4CV	1.11	7.82	0.13	0.09	-0.82	-0.40	0.13	0.01	-0.14	0.07	0.33	0.32	-0.18	0.15	0.04	0.15
HZS	PH4CV	-3.71	0.12	0.42	0.12	0.06	-2.43	-0.11	-0.27	-0.22	-0.13	-0.11	0.25	-0.20	0.09	-1.89	-0.05
5213	PH4CV	1.18	1.77	-0.04	-0.05	1.30	-0.29	0.04	0.55	-0.35	0.17	-1.02	1.54	0.73	0.45	-0.81	-0.09
CN165	PH4CV	7.39	-0.33	1.86	-0.07	1.69	-0.68	-0.56	0.30	0.00	0.27	-0.44	0.54	0.41	-0.30	1.14	0.04
CN4379	PH4CV	11.54	10.33	1.12	-0.08	0.31	-1.17	0.00	0.87	-0.19	-0.07	-0.32	1.19	0.00	0.08	0.33	0.03
Qi318	PH4CV	3.99	3.56	0.53	-0.06	-0.35	0.44	-0.40	-3.45	-0.34	0.01	-0.25	2.94	-0.58	0.70	0.72	0.08
YFH	PH4CV	-5.88	-2.04	-0.37	0.02	-0.64	-2.56	0.00	-1.08	0.16	0.18	-0.52	-0.59	0.62	0.10	2.43	0.06
P007	PH4CV	-1.31	2.32	-0.87	-0.03	-1.64	-0.24	0.32	-2.37	-0.06	-0.09	-2.48	-1.01	0.28	0.17	-1.12	-0.05
F349	PH4CV	0.52	4.14	-0.48	0.02	-1.64	-0.54	0.30	-0.34	0.33	0.09	-1.03	1.21	0.17	0.50	2.61	-0.08
P25	PH4CV	10.92	9.82	1.61	-0.07	0.62	0.59	0.44	-0.45	0.09	0.46	-1.56	2.56	0.51	0.11	0.04	0.02
Qi205	PH4CV	-5.95	-2.01	-0.70	0.06	0.27	-0.90	0.38	-0.79	-0.06	0.08	-0.21	-0.06	-0.39	0.79	-0.67	0.00
CA375	PH4CV	2.52	-0.41	0.65	-0.02	-0.02	-1.91	-0.13	0.14	0.11	-0.10	-0.84	-0.05	-0.15	0.10	-0.93	0.06
Jing501	PH4CV	8.06	2.81	0.55	-0.04	0.60	0.49	0.05	-2.27	0.16	0.13	-0.80	1.27	0.67	-0.29	-0.67	0.06
Jing24	PH4CV	-8.39	-1.24	-0.77	0.03	-1.13	-0.97	-0.03	0.61	-0.18	0.39	0.33	-0.88	-0.48	0.33	-0.09	0.02
Jing89	PH4CV	3.98	3.38	0.17	0.09	-0.74	-1.35	-0.24	-1.88	0.01	0.41	-0.41	2.10	-0.90	0.06	2.12	0.03
Jing572	PH4CV	-0.31	2.29	-0.66	0.00	-0.23	-0.70	0.01	-1.56	0.09	-0.16	-0.78	0.97	0.09	0.32	0.36	-0.01
NH60	PH4CV	17.79	5.74	0.12	0.06	1.77	0.44	0.10	0.05	0.43	0.16	0.21	-1.38	1.57	-0.08	-0.04	0.05
Jing724	PH4CV	15.33	8.94	0.55	0.11	2.66	0.47	-0.22	2.53	0.31	0.24	0.59	-1.55	1.58	-0.36	0.03	0.20
Jing92	PH4CV	-0.34	0.71	-0.55	0.02	-0.49	-1.74	-0.22	-2.26	-0.27	0.09	1.56	0.47	0.29	0.36	2.14	0.03
Jing725	PH4CV	11.72	5.33	0.00	-0.04	1.53	1.46	-0.50	-0.19	0.04	0.26	1.44	-1.51	0.21	0.37	0.83	0.06
Aijing525	PH4CV	0.61	-2.11	1.15	-0.21	2.70	-0.69	-0.41	-0.71	-0.50	-0.27	-1.80	-4.75	0.53	0.18	-1.66	-0.05
C103	PH4CV	-8.44	-3.62	-0.30	0.02	0.45	-2.16	-0.12	-1.28	0.16	0.13	0.59	0.95	0.16	0.01	1.59	-0.10
Zheng58	PH4CV	-1.28	-0.24	0.44	0.01	0.45	0.50	0.32	-0.64	-0.40	-0.42	0.04	-0.19	-0.34	-0.36	-2.60	0.11
XL21	PH4CV	-5.07	-5.02	0.92	0.05	-0.41	0.76	-0.53	0.20	-0.61	0.11	-0.06	0.31	0.51	-0.90	-1.20	-0.13
H2671	PH4CV	1.82	-0.29	0.24	0.05	-0.43	1.22	0.13	1.51	0.14	-0.11	0.18	-1.61	-0.51	-0.13	-1.08	0.00
H2671	PH4CV	1.82	-0.29	0.24	0.05	-0.43	1.22	0.13	1.51	0.14	-0.11	0.18	-1.61	-0.51	-0.13	-1.08	0.00
CL11	PH4CV	1.92	1.17	-0.07	0.07	0.21	-0.29	-0.01	-0.54	0.19	-0.05	-0.22	-1.85	-0.45	0.01	0.04	0.13
NG5	PH4CV	-0.01	1.34	1.71	-0.16	0.09	1.50	-0.43	-1.09	-0.03	0.61	-1.17	1.61	-0.30	0.77	1.39	-0.03
HD568	PH4CV	-5.21	-1.08	-0.85	-0.08	-0.99	0.59	1.29	-2.12	-0.39	-0.24	0.46	0.38	-0.31	0.05	0.10	-0.15
11DM124	PH4CV	15.10	0.78	1.46	0.11	2.73	0.79	-0.76	-2.27	0.30	0.42	-0.27	2.71	1.30	0.02	3.82	0.11
CA616	PH4CV	-33.60	-8.90	-4.63	-0.46	-6.79	2.21	0.17	-2.86	-1.48	-0.45	-1.10	2.19	-2.12	-0.30	-5.39	-0.47
Ji6003	PH4CV	-3.33	-0.19	-0.01	-0.06	-0.77	0.92	-0.20	0.55	0.09	-0.06	-0.27	-1.49	0.17	0.19	-1.56	-0.03
Ming2325	PH4CV	-1.58	-3.49	0.05	-0.10	1.76	0.37	-0.43	-0.66	0.00	0.21	-0.60	-0.40	0.04	-0.73	-0.66	0.12
S5137	PH4CV	-0.61	-8.32	-0.49	-0.20	-0.90	-1.59	0.24	-3.44	-0.37	0.01	-0.67	4.17	0.68	-0.93	0.86	-0.28
F2001	PH4CV	1.29	0.31	2.34	0.38	7.87	-1.66	0.06	4.04	0.22	0.71	0.01	0.95	1.85	-2.04	2.04	0.09
20143	PH4CV	0.02	-0.37	0.04	-0.01	0.25	-1.38	-0.47	-1.67	-0.17	0.20	-0.29	-0.43	0.22	-0.01	0.20	0.10
Cheng60	PH4CV	-7.33	-4.17	-0.97	-0.11	0.25	0.85	-0.29	-0.06	-0.11	0.33	-1.25	-3.41	-0.23	-0.68	-3.28	-0.27
Cheng53	PH4CV	-0.40	-7.93	-0.63	-0.07	0.65	-3.46	1.19	-3.03	-0.44	-0.40	-3.78	3.11	1.34	-1.06	-0.39	-0.31
Chong72	PH4CV	-10.06	-5.18	0.70	0.05	0.85	-0.99	0.59	-2.67	0.12	0.01	0.69	1.73	0.84	-1.01	0.89	-0.09
NP01200	PH4CV	5.22	-2.86	0.62	0.37	1.50	-1.12	-0.42	0.00	-0.04	-0.26	0.08	0.10	0.27	-0.65	0.77	0.14

PH6AT	PH6WC	-6.10	-2.04	-1.66	-0.16	-0.35	-0.70	-0.67	-1.12	-0.30	-0.22	-0.44	0.31	-0.43	0.14	-0.42	-0.20
PHB1M	PH6WC	18.37	6.33	2.28	0.23	1.32	-1.05	0.34	2.44	1.11	0.51	1.99	-1.78	1.30	-0.15	3.23	0.40
ZhongM_8	PH6WC	-0.91	0.67	0.03	-0.08	-1.02	-0.67	0.16	-0.37	-0.15	-0.27	-0.32	0.32	0.43	0.24	-0.98	0.09
L237	PH6WC	1.73	-0.77	0.89	-0.08	1.01	-0.41	-0.14	-0.42	-0.71	0.67	-0.52	2.38	-1.30	-0.84	1.15	-0.09
Ying64	PH6WC	2.35	1.65	0.45	0.06	-1.00	0.54	-0.11	0.56	0.09	0.20	0.86	0.57	-0.49	0.26	1.08	0.01
Dan891	PH6WC	-4.84	-5.37	-2.09	0.08	0.27	-0.36	0.77	-5.66	-0.28	0.64	0.85	2.79	0.50	0.15	-0.65	-0.08
DG11A	PH6WC	3.30	2.10	-0.09	-0.02	0.98	0.77	-0.65	-0.50	0.03	-0.02	0.27	0.60	1.20	0.32	-0.12	-0.04
Fu746Mu	PH6WC	-0.53	4.32	-0.33	-0.17	-3.19	2.45	-0.22	2.88	-0.51	0.41	-0.91	0.99	-0.49	-0.43	-1.40	0.09
Dong46	PH6WC	2.00	-0.78	0.09	-0.03	0.24	0.98	0.43	-0.31	-0.10	-0.22	-0.69	0.17	-0.40	-0.20	-1.06	-0.16
Dong237	PH6WC	1.60	3.45	-1.30	0.05	-1.05	-0.31	0.10	1.38	-0.13	1.62	0.71	0.03	0.24	0.20	-1.14	0.00
Hai268	PH6WC	5.01	-1.87	0.46	-0.24	0.91	-0.36	0.76	-0.49	0.13	-0.81	-0.29	0.54	0.11	-0.11	0.21	0.09
1134	PH6WC	-17.41	-1.92	1.63	-0.13	1.04	3.14	-0.50	3.57	-0.38	1.33	1.49	1.82	-0.37	-0.86	-1.40	0.07
K10	PH6WC	-2.57	-0.19	-0.11	-0.05	-1.21	0.45	-0.36	-0.08	0.10	-0.16	-0.21	-0.62	0.08	-0.35	-0.41	-0.12
LK11	PH6WC	7.44	3.53	0.82	-0.04	2.06	0.24	-0.51	2.31	0.05	0.61	-0.25	0.03	-0.48	-0.29	0.50	0.16
KL3	PH6WC	-2.73	-2.94	0.71	-0.11	-1.38	-1.46	-0.60	-0.28	-0.27	-0.25	0.28	0.57	-1.34	0.16	-1.71	-0.04
KL4	PH6WC	0.20	3.66	-0.83	0.03	1.24	0.06	-0.37	-0.71	-0.05	0.56	-0.15	0.98	0.10	0.46	-0.83	-0.14
Long53	PH6WC	3.42	1.32	0.21	-0.04	0.12	-0.96	-0.27	-1.35	-0.22	0.10	-0.32	2.32	0.00	0.33	1.95	0.00
Fu706	PH6WC	7.23	1.19	-0.07	-0.06	-1.21	1.78	0.37	0.57	-0.10	-0.04	0.21	2.70	0.66	-0.54	-1.79	0.02
He344	PH6WC	-1.93	-5.33	0.74	0.00	-0.53	0.31	-0.18	0.04	-0.07	0.27	1.14	0.45	0.77	0.07	0.78	-0.06
8941	PH6WC	0.46	2.16	0.68	0.01	0.55	0.11	-0.14	-0.07	0.06	0.18	0.39	1.01	0.04	0.12	0.95	-0.08
Dong6002	PH6WC	4.06	6.99	-0.11	-0.06	-0.53	0.33	-0.12	-0.76	0.00	0.29	0.61	0.75	-0.65	-0.49	0.10	-0.08
Ji846	PH6WC	8.45	7.33	1.69	0.32	-0.04	-0.37	0.05	-1.13	0.17	-0.11	0.31	2.74	-0.13	0.05	1.89	0.04
KL2	PH6WC	-1.69	-2.13	-0.34	-0.05	-0.26	-0.06	-0.45	-2.13	0.06	-0.25	-0.08	0.72	-0.36	-0.65	-0.36	-0.12
KL6	PH6WC	-0.22	5.41	-1.35	-0.13	-3.73	0.06	0.13	-2.80	0.08	0.15	-0.03	0.27	-0.79	-0.38	-1.17	-0.17
D3_1	PH6WC	9.96	-2.16	1.56	-0.02	1.36	-0.02	0.39	0.09	0.05	-0.38	-0.68	1.40	0.29	0.45	0.20	0.10
D5_2	PH6WC	-3.71	4.10	-2.79	0.10	-0.59	-0.77	-0.20	-2.17	-0.02	0.39	0.73	-0.93	-0.66	0.57	-1.45	0.13
HR30	PH6WC	2.65	2.06	0.29	-0.16	-0.68	-0.40	-0.18	1.42	0.08	0.27	0.54	0.56	-0.08	0.41	-1.73	0.03
KWS10_73	PH6WC	4.42	5.43	-0.57	-0.05	-1.36	0.21	-0.06	0.58	0.38	0.46	-0.44	-1.68	-0.05	-0.16	2.31	0.01
KWS49	PH6WC	2.20	4.28	-0.77	0.10	0.04	-0.26	-0.12	-1.04	0.07	0.07	-0.71	0.33	-0.24	0.21	-0.11	0.00
Jia33	PH6WC	-8.42	-2.48	-1.09	-0.06	-0.26	0.66	-0.09	-0.12	0.05	0.24	-0.45	-1.40	-0.18	-0.42	-1.13	-0.14
Jia28	PH6WC	2.20	0.75	-0.07	-0.15	0.25	-0.71	-0.06	0.37	-0.06	-0.18	-1.45	-0.83	0.30	-0.64	-2.47	0.12
KW5G321	PH6WC	14.90	3.54	1.93	0.21	1.97	-0.68	0.11	0.57	0.01	0.27	0.14	0.48	1.41	-0.19	0.91	0.16
KW1A139	PH6WC	-1.00	4.62	0.27	0.05	-1.30	-0.39	0.23	0.72	0.17	-0.11	0.03	-1.43	-0.54	-0.14	-0.51	0.03
H1208	PH6WC	-1.96	5.50	1.13	-0.05	-1.85	1.74	-0.11	0.64	0.01	0.11	-1.31	-0.60	-0.16	0.77	-0.03	-0.03
L203	PH6WC	-2.07	1.16	0.34	0.04	-0.21	-0.34	-0.12	0.05	0.19	0.32	0.05	1.09	-0.60	0.12	0.96	-0.19
81162	PH6WC	2.48	-1.43	1.48	-0.06	1.31	0.51	0.08	1.68	0.00	-0.12	-0.98	-0.74	-0.75	0.38	0.00	0.11
8638	PH6WC	3.61	4.09	1.66	0.02	-0.08	0.34	-0.36	1.24	-0.32	0.03	-0.46	1.44	0.36	0.00	0.27	-0.07
833	PH6WC	-1.25	1.57	0.09	0.01	-0.96	-0.50	0.19	0.38	0.01	0.30	-0.63	0.12	-0.15	0.33	-0.37	-0.01
9F592	PH6WC	-0.02	-4.49	-0.15	-0.06	-1.15	-0.19	-0.14	1.34	-0.38	0.02	0.19	-0.94	-0.44	-0.70	-1.55	-0.14
6F576	PH6WC	3.93	4.07	1.56	-0.02	0.39	-0.62	-0.63	1.09	0.28	-0.10	0.55	-0.26	0.52	-0.42	0.51	0.01
KL613	PH6WC	1.51	-0.06	-0.04	-0.07	0.24	0.40	-0.35	1.25	-0.73	0.09	-1.98	-0.96	-0.67	-0.10	-2.10	-0.21
385_1	PH6WC	-0.93	-3.86	-0.48	0.10	-0.37	-0.21	0.51	1.91	0.23	0.22	0.02	-1.73	-0.24	0.12	0.47	-0.08
HA25	PH6WC	6.00	4.39	3.23	-0.05	-1.00	-0.07	-1.30	2.58	-0.02	0.25	-0.62	-2.67	-0.49	-0.72	-0.46	0.02
KL45	PH6WC	-1.69	-3.98	0.89	-0.05	1.99	-1.07	0.10	0.92	-0.38	0.22	-0.04	0.50	-0.39	-0.53	0.31	-0.09

Ji63	PH6WC	0.04	1.47	-0.74	-0.10	-0.52	1.48	-0.22	-1.15	0.53	0.17	-1.14	-1.70	0.69	-0.13	-0.06	-0.06
Men14	PH6WC	-7.77	-3.72	-1.23	-0.10	0.47	-0.95	0.07	-2.68	-0.06	0.40	0.16	0.85	0.02	0.09	0.39	-0.08
Xi14	PH6WC	0.95	-0.56	-0.15	0.02	1.17	-0.44	0.38	-0.58	0.04	-0.05	0.85	-0.23	0.18	-0.07	-0.31	0.07
Mo17	PH6WC	-0.27	-4.67	0.59	-0.08	0.67	-0.49	0.02	-0.14	-0.28	0.09	-0.28	0.15	-0.01	0.00	-1.56	0.01
ZaC546	PH6WC	-0.61	-1.40	0.49	-0.14	-0.88	0.23	0.18	1.13	-0.32	-0.16	-1.26	-2.26	-0.16	0.21	-2.78	0.04
70_104	PH6WC	-3.19	-4.14	-1.12	0.03	0.22	-0.52	-0.01	-1.24	0.24	0.35	-0.76	0.27	-0.36	-0.75	1.95	0.11
L105	PH6WC	3.73	3.43	1.00	-0.13	-0.10	0.39	0.08	1.86	-0.01	-0.18	-1.86	0.75	0.06	0.13	-0.50	-0.06
Zi330	PH6WC	1.67	0.91	0.03	-0.04	0.59	0.07	-0.84	0.45	-0.17	-0.27	0.02	0.89	0.28	0.30	0.08	-0.11
446	PH6WC	-2.76	-3.90	0.42	-0.04	0.61	0.23	-0.50	2.41	0.03	-0.09	-0.34	0.03	-0.74	0.24	0.07	-0.07
Ji853	PH6WC	2.28	2.93	0.63	0.00	-0.80	2.03	0.28	0.30	-0.15	0.13	0.07	0.89	0.91	-0.27	0.60	-0.08
434	PH6WC	6.96	4.72	1.78	0.08	0.48	-0.74	-0.12	0.38	0.01	0.28	-0.80	-0.54	0.46	0.01	1.17	0.04
4F1	PH6WC	-4.18	-4.44	-0.12	-0.03	1.04	0.09	-0.03	-0.63	-0.05	0.02	-0.70	1.18	0.88	-0.72	0.21	0.05
444	PH6WC	-0.50	1.32	0.09	-0.02	-0.11	-0.42	-0.19	-0.02	0.02	-0.02	-0.20	-0.15	-0.31	0.03	0.49	-0.05
Ji002	PH6WC	-4.17	-3.28	-1.21	0.03	1.20	0.05	0.13	-0.44	-0.09	0.08	0.06	-1.52	-0.27	0.01	-0.20	-0.18
S8_101	PH6WC	-1.02	-4.34	-0.67	0.16	0.39	-1.42	0.50	-0.33	-0.09	-0.04	2.25	-1.46	-0.25	-0.70	-0.70	-0.14
7922	PH6WC	10.19	3.36	0.84	0.17	1.20	-0.66	0.25	-0.95	0.49	0.60	0.61	-0.47	-0.07	0.26	1.17	0.14
7884_7Ht	PH6WC	7.81	0.06	-0.81	0.11	0.71	0.79	-0.05	-1.69	0.42	0.23	0.59	0.49	0.92	1.43	5.66	-0.03
96478	PH6WC	3.05	2.20	-0.15	0.14	1.03	-0.71	0.38	0.76	0.05	-0.21	0.69	-0.19	0.44	0.12	1.86	0.02
Ji992	PH6WC	0.98	-1.97	0.48	-0.04	-0.73	-0.24	0.04	1.69	-0.17	0.15	-0.21	-1.54	0.50	0.10	-0.83	0.04
Ji1037	PH6WC	-1.63	-3.30	0.25	-0.03	0.18	0.53	-0.12	-2.14	-0.33	0.29	0.04	1.34	0.42	-0.10	0.93	0.03
Si_287	PH6WC	5.95	-0.85	0.64	0.04	0.61	0.13	0.69	0.84	-0.14	0.47	0.40	-0.20	-1.01	-0.10	-1.78	0.14
W9706	PH6WC	-2.66	3.54	0.52	-0.06	0.05	0.74	-0.29	-0.13	-0.47	-0.23	-0.82	0.65	-0.38	0.66	-2.76	0.08
8902	PH6WC	0.63	4.18	0.10	0.09	0.81	-0.75	0.37	0.26	0.36	-0.06	-0.44	-0.37	0.09	0.21	1.30	-0.04
Cheng351	PH6WC	2.78	0.17	-0.15	0.11	0.43	-0.80	-0.17	-0.84	-0.07	-0.08	1.33	-0.95	-0.15	0.11	0.30	0.12
D22	PH6WC	0.39	0.52	0.96	-0.08	-0.15	-0.69	-0.10	0.80	-0.16	0.02	-1.22	-0.86	-0.15	-0.15	-0.99	0.01
KX	PH6WC	6.38	-1.25	1.42	0.00	1.21	-0.78	-0.59	1.94	0.48	0.40	-0.44	1.65	0.24	-0.13	2.68	-0.08
Ji046	PH6WC	-12.44	-4.79	-0.27	-0.10	0.92	-1.39	-0.11	-0.18	0.47	-0.37	-1.10	-2.40	-0.32	-0.53	-1.80	0.03
Dan598	PH6WC	3.09	2.07	0.03	-0.02	0.29	0.39	-0.06	-0.78	-0.11	-0.11	-0.13	0.30	0.46	0.12	-0.42	-0.04
634150	PH6WC	-0.59	-2.08	-1.75	-0.09	-1.50	-3.13	-0.34	-2.02	0.05	-0.41	-1.35	-0.11	-1.47	-0.20	-1.62	-0.09
TM	PH6WC	7.56	3.43	-0.14	-0.06	0.43	1.74	0.14	-0.32	0.08	0.05	-0.57	-0.22	-0.02	-0.31	-0.76	-0.09
M407	PH6WC	-15.93	-3.92	-1.03	-0.17	-1.14	-1.18	0.10	-0.62	-0.45	-0.58	-0.73	1.03	-1.00	-0.80	0.31	-0.06
F62	PH6WC	6.64	3.75	-1.38	-0.07	0.39	0.45	0.30	-2.08	-0.06	0.09	1.13	0.98	-0.75	0.32	0.43	0.12
Dan340	PH6WC	-3.66	-3.47	0.30	0.05	0.33	0.80	-0.78	-0.69	-0.36	-0.18	-1.29	0.54	0.20	0.05	0.50	0.05
Si428	PH6WC	-3.96	-5.90	-0.25	-0.15	-0.09	0.53	0.24	-2.32	0.44	0.62	-1.42	0.59	0.96	-0.39	0.86	-0.11
Cheng18	PH6WC	2.76	-0.21	1.20	0.03	0.21	0.41	-0.08	0.97	-0.03	-0.27	-0.21	0.70	-0.79	0.40	0.69	0.02
Si144	PH6WC	5.19	3.54	0.07	-0.03	-1.05	-0.17	-0.33	0.32	-0.24	0.06	-0.26	-0.25	-0.03	-0.14	-0.09	0.03
HYM	PH6WC	1.34	0.10	-0.18	-0.08	-0.77	-1.54	-0.48	-0.96	-0.15	-0.41	-0.63	0.93	-0.69	0.48	0.95	-0.01
Lv28	PH6WC	-7.43	-5.35	-0.26	0.05	0.54	0.79	0.20	-1.59	-0.01	0.53	0.26	-0.27	-0.07	-0.50	-0.91	-0.04
E28	PH6WC	0.88	-4.41	-0.19	-0.12	0.18	-0.88	-0.22	0.09	-0.27	-0.63	-1.60	1.10	0.40	0.03	-0.07	-0.17
Mo17Ht	PH6WC	1.51	-3.27	0.41	-0.01	0.61	0.87	0.21	-1.63	-0.24	-0.27	-0.32	1.79	-0.18	-0.45	0.65	-0.06
Shen5003	PH6WC	-3.92	-7.55	-0.44	0.10	1.56	0.30	-0.01	-1.44	-0.10	-0.06	0.10	-0.72	0.52	0.22	1.11	0.12
KYC8605	PH6WC	9.06	3.77	-0.43	0.03	-0.19	0.67	0.24	0.98	0.10	0.43	0.32	-0.28	0.74	0.34	1.08	-0.02
7_61	PH6WC	1.03	-1.29	0.00	0.01	0.98	0.83	0.19	0.70	0.25	-0.15	-0.37	-0.75	-0.10	-0.45	-0.90	-0.02
Liao2345	PH6WC	5.50	-2.75	0.47	0.13	1.40	-0.23	-0.35	1.92	0.46	0.55	0.61	-0.84	0.86	0.59	2.82	0.12

Q1261	PH6WC	4.35	4.00	0.08	0.01	0.32	-1.00	0.03	-0.18	-0.03	-0.10	-0.66	-1.71	-0.35	0.69	-1.58	0.00
Shen137	PH6WC	0.77	1.10	1.02	0.18	1.64	-1.32	-0.27	-0.28	-0.01	0.45	0.05	1.35	0.47	-0.13	1.57	-0.02
Dan9046	PH6WC	-18.51	-2.45	-0.97	-0.12	3.85	1.61	-0.75	-0.60	-0.29	0.45	-0.17	-1.23	1.27	-1.27	-0.34	0.16
8112	PH6WC	-4.83	-2.23	0.15	0.18	-0.38	-1.02	0.47	-0.42	0.31	-0.15	0.34	0.24	0.87	-0.09	2.23	0.10
Liao1412	PH6WC	-0.09	-2.76	-0.86	-0.07	-0.04	0.31	0.29	-2.05	0.30	0.21	0.46	-1.32	0.10	-0.69	-0.04	-0.13
LD175_1	PH6WC	-1.26	0.47	1.76	-0.09	-0.38	-0.45	0.45	1.38	0.03	-0.15	-0.36	-0.28	0.22	0.45	2.52	-0.04
LD61	PH6WC	16.46	15.03	0.70	0.03	1.37	1.43	0.23	1.48	0.20	0.10	1.29	-2.33	0.78	1.12	0.13	0.30
D26	PH6WC	-0.24	-0.32	-0.12	0.03	0.63	-1.47	0.23	-1.29	0.09	-0.07	0.09	0.23	-0.24	0.30	1.57	-0.03
Dan3130	PH6WC	-6.40	-2.13	-1.71	-0.22	-0.19	0.27	0.29	-1.39	-0.40	-0.22	-0.81	4.39	0.03	0.27	-1.44	-0.23
A801	PH6WC	-3.14	-5.90	0.28	0.00	0.94	-1.49	-0.25	-1.53	0.05	0.09	-0.17	-0.79	-0.08	-0.73	0.89	0.07
Liao68	PH6WC	-5.38	-7.65	0.12	0.14	0.61	-0.88	-0.26	-0.21	-0.06	0.29	0.99	0.82	0.04	-0.08	2.79	0.01
LIao3180	PH6WC	8.29	2.20	0.71	0.17	0.78	-1.31	-0.15	-0.50	0.33	0.24	0.58	-0.20	0.23	0.21	2.19	0.15
K12	PH6WC	7.74	6.53	-0.49	0.05	-0.03	-2.60	0.00	-0.87	-0.10	-0.08	-0.46	-0.53	-0.27	0.31	0.05	0.13
Shen151	PH6WC	5.01	4.83	0.38	0.14	0.05	0.56	0.55	-2.01	0.04	-0.47	0.47	1.73	0.46	0.53	2.28	0.26
Dan717	PH6WC	2.07	5.96	0.76	-0.03	1.29	-0.84	0.03	-1.47	-0.26	0.01	-0.45	-0.35	0.16	0.35	0.66	0.04
Liao8478	PH6WC	0.84	-0.66	-0.12	0.10	2.16	-0.12	-0.05	-2.13	0.06	0.23	0.42	-0.48	1.16	0.05	1.41	-0.02
Zheng22	PH6WC	-0.31	-0.31	-0.19	-0.17	-0.60	0.48	-0.28	-1.07	-0.56	-0.11	-0.43	1.39	0.16	-0.50	-3.12	-0.20
Zhong106	PH6WC	2.00	-0.68	-0.23	-0.13	0.61	0.17	-0.09	-0.16	-0.15	-0.10	-2.22	-0.77	0.15	-0.48	-1.30	-0.12
Liao3162	PH6WC	-7.05	1.70	0.08	0.20	0.69	1.53	0.39	5.15	0.35	-0.02	0.08	-0.96	0.94	0.00	2.85	0.23
PH09B	PH6WC	24.00	8.17	2.76	0.06	4.48	-0.50	0.17	-3.84	0.08	0.29	0.16	0.49	2.86	0.68	2.76	0.08
PHPMO	PH6WC	-24.39	-11.03	-2.60	-0.40	-2.80	-1.21	0.17	-4.91	-1.24	-0.04	-1.89	2.00	-0.60	-0.74	-2.95	-0.37
Dan988	PH6WC	-2.77	-6.86	-0.35	0.02	-0.36	-0.38	0.05	-0.03	0.11	0.18	-0.79	-1.71	0.69	-0.25	0.82	-0.02
DanT138	PH6WC	1.76	0.75	-0.06	-0.08	-0.34	-1.10	0.09	-0.57	-0.07	-0.14	-0.82	0.79	0.77	-0.29	1.64	-0.06
Liao6082	PH6WC	-2.53	-3.68	-0.55	0.13	-0.24	-0.74	0.57	-0.95	0.20	0.02	-1.04	-0.11	0.41	0.18	-0.19	0.04
63792	PH6WC	2.11	3.16	1.87	0.16	0.05	-0.97	0.13	2.34	0.36	-0.01	-1.23	-0.59	1.33	0.05	1.66	0.15
7017	PH6WC	5.96	-1.91	-0.85	-0.20	0.42	-0.47	-0.26	1.07	-0.60	0.11	-1.53	-1.85	0.09	0.52	-3.85	-0.25
Dan99C	PH6WC	4.02	3.57	-0.13	-0.04	0.30	0.47	0.42	0.53	-0.18	0.04	0.39	0.36	0.45	0.12	0.62	-0.05
Shen3336	PH6WC	-1.06	-0.18	-0.29	-0.16	0.80	-0.62	-0.36	-1.37	-0.36	-0.10	-0.40	0.69	0.67	0.04	0.62	-0.23
Shen3265	PH6WC	-1.56	2.77	-1.12	-0.02	0.34	0.06	-0.15	-1.19	0.25	0.02	-0.86	-1.10	-0.07	-0.08	1.03	-0.14
T98103_1	PH6WC	6.61	5.21	-0.19	0.10	0.03	-1.19	0.07	-0.09	0.18	0.07	0.11	-1.64	-0.29	1.47	0.02	0.10
Dan360	PH6WC	0.05	0.99	0.64	0.05	-0.67	2.96	0.57	0.01	0.01	-0.19	0.75	0.00	0.54	-0.34	0.05	0.15
CL143	PH6WC	-7.24	-0.81	-2.51	-0.01	1.32	0.54	0.84	-1.86	-0.74	0.18	-0.25	-4.20	-0.16	0.41	-3.25	-0.25
CH382	PH6WC	5.76	3.42	-1.37	0.04	-1.36	0.19	-0.45	-2.43	-0.26	-0.04	0.05	-1.31	-0.85	0.64	0.07	-0.03
PH6JM	PH6WC	10.77	4.27	1.69	0.15	-0.10	0.95	-0.30	0.59	0.60	0.32	-0.69	-0.44	0.35	0.67	4.10	0.23
Dan6263	PH6WC	-0.09	0.63	-0.44	0.03	0.86	-0.34	0.31	-1.29	-0.18	-0.10	0.44	-0.05	0.37	-0.22	-0.90	-0.05
C260	PH6WC	-1.87	2.30	-0.49	0.15	1.11	-1.37	0.41	1.30	0.35	-0.39	0.43	1.52	-0.48	0.13	0.60	0.21
C168	PH6WC	2.27	1.29	-0.26	-0.05	-1.04	-0.13	-0.21	-2.00	-0.20	0.04	0.10	1.05	0.15	0.24	0.89	-0.12
Liao7980	PH6WC	-4.52	-0.59	-0.57	0.10	0.36	-0.92	0.47	0.03	-0.02	-0.20	0.58	-0.44	-1.11	-0.85	0.25	-0.03
F12	PH6WC	4.37	3.25	0.44	0.09	-0.59	0.31	0.44	0.93	0.12	-0.32	0.48	-0.16	0.77	-0.10	-1.09	0.11
K0325	PH6WC	-1.81	-1.57	-0.83	0.05	-0.46	0.76	0.10	-2.79	0.00	-0.08	0.30	0.73	-0.65	0.24	-0.02	0.03
2511A	PH6WC	1.91	-1.16	-0.28	-0.08	-1.30	0.06	-0.21	0.68	-0.33	0.18	-0.10	-0.17	-0.66	0.00	-0.02	-0.23
CH382	PH6WC	2.76	2.14	0.50	0.06	1.26	-0.39	0.08	0.10	0.21	0.17	0.14	0.86	0.66	0.04	1.66	0.10
Z3_87	PH6WC	-8.89	-5.18	-1.24	-0.14	-1.20	-0.91	-0.26	-2.04	-0.24	-0.10	-0.50	0.21	-0.26	-0.38	-0.97	-0.08
M60	PH6WC	-19.74	-2.65	-0.56	-0.04	-0.28	-0.38	-0.04	-2.81	-0.22	0.03	-0.29	0.56	0.20	-0.33	0.00	-0.05

S121	PH6WC	-32.58	-16.22	-2.87	-0.40	-3.45	-1.55	-0.07	-4.15	-1.32	0.13	-0.93	3.47	-2.09	-0.63	-3.89	-0.54
P2237	PH6WC	-1.64	-4.57	0.39	0.12	1.04	0.46	0.24	-1.59	0.05	0.06	0.62	-0.34	0.16	-0.07	1.18	0.06
K3841	PH6WC	4.80	8.44	0.54	0.00	0.10	-0.41	-0.13	-0.11	-0.04	0.22	0.77	0.89	-0.47	0.21	0.76	0.14
SD7928	PH6WC	2.21	0.97	1.71	0.10	0.31	-1.49	0.12	-0.86	-0.05	0.09	0.40	1.45	0.35	0.39	2.48	0.04
SD8738	PH6WC	-8.00	-0.57	-0.03	-0.06	-1.70	-1.67	-0.59	-1.58	-0.54	-0.30	-0.72	1.66	-0.56	-0.10	-0.78	-0.09
Shen3117	PH6WC	-3.11	-2.99	-0.53	-0.06	0.58	-0.47	-0.08	-0.43	-0.20	0.39	-0.25	0.71	-0.52	-0.53	1.23	-0.18
Dan1133	PH6WC	-5.81	-4.13	0.33	0.16	0.36	-0.87	0.42	0.99	0.33	-0.24	0.30	-0.62	0.22	-0.88	-0.11	0.01
Dan37	PH6WC	0.68	-1.44	0.09	0.02	-0.29	1.71	-0.19	0.31	0.21	0.05	-0.13	-0.61	-0.17	0.25	0.46	-0.04
M03	PH6WC	-6.54	-4.65	-0.42	0.02	0.52	-0.47	-0.05	-1.28	0.34	-0.35	-0.32	1.06	-0.06	0.01	0.12	0.08
M5972	PH6WC	-3.35	-5.98	-1.14	0.07	-1.03	-0.03	0.00	-2.14	0.02	0.28	0.88	-0.07	-0.59	-0.28	-0.39	-0.15
Ming84	PH6WC	-4.55	-0.30	-0.14	-0.03	-0.28	0.53	-0.17	0.05	0.07	0.04	-0.28	-0.31	0.57	0.42	1.50	0.15
Ming71	PH6WC	0.46	-0.24	0.03	0.01	1.01	-1.17	-0.18	-0.02	0.09	0.21	0.03	-0.16	-0.52	0.04	0.58	0.04
T0278	PH6WC	-1.87	-4.70	-0.10	0.04	0.09	-0.22	0.27	1.72	-0.10	-0.31	1.05	-1.04	-0.01	-0.56	0.08	0.01
TieT0403	PH6WC	0.59	0.16	1.03	-0.03	-0.73	1.91	-0.04	1.43	0.20	0.30	-0.67	-0.02	0.24	-0.36	-0.28	0.03
M53	PH6WC	-12.99	-2.10	-1.75	-0.19	-2.83	-1.08	-0.78	-3.70	-0.34	-0.27	-0.53	0.56	-0.46	-0.17	-1.80	-0.13
S127	PH6WC	-3.74	-4.56	0.06	0.10	0.43	-0.13	-0.35	1.27	-0.12	0.24	0.78	0.58	1.07	-0.77	-0.06	0.03
Liao8821	PH6WC	-3.13	-6.95	0.64	0.09	-0.84	-1.38	-0.14	1.30	0.11	0.08	0.16	1.12	-0.06	0.26	1.88	0.08
Shen391	PH6WC	-3.30	2.31	-1.44	-0.05	0.22	-1.35	-0.15	-1.35	0.02	-0.05	-1.11	-0.89	0.37	0.23	-0.17	-0.07
Shen8078	PH6WC	-3.77	-4.47	-0.15	-0.04	0.55	0.53	0.12	-2.09	-0.28	-0.42	-0.56	0.13	0.29	0.24	0.04	-0.10
Tie84	PH6WC	-3.16	-1.66	0.12	-0.35	-0.30	-0.80	0.08	-1.94	-0.14	0.26	-0.88	-0.23	0.52	-0.13	-1.59	-0.07
Lv9W	PH6WC	5.81	5.21	-0.28	-0.05	-0.41	0.42	0.22	-0.91	-0.28	-0.50	-0.67	1.58	-0.90	0.05	-0.70	0.14
9137	PH6WC	-1.12	4.63	-0.24	0.05	-1.17	0.64	-0.01	-0.88	0.09	0.04	-0.24	0.07	-0.32	0.36	0.81	0.09
391	PH6WC	2.12	-2.53	-0.57	0.04	0.51	0.25	0.68	-0.01	-0.08	-0.35	1.05	0.48	0.04	0.02	-0.99	0.03
XKZ101_1	PH6WC	2.05	0.94	1.30	-0.01	0.52	0.35	-0.61	3.22	0.11	0.22	-0.43	-0.34	0.04	-0.01	-0.76	-0.02
XKZA34	PH6WC	1.46	0.29	0.60	-0.02	-1.02	-0.46	-0.05	-0.32	-0.39	-0.40	-0.35	-0.06	0.41	-0.08	-0.94	-0.11
XKZ167_1	PH6WC	-2.79	3.94	-0.26	-0.02	0.40	-0.03	-0.64	0.31	0.22	0.32	-0.45	-1.41	0.05	0.26	-0.14	-0.07
GLMo17	PH6WC	0.09	-2.93	-0.61	-0.14	-1.02	-0.05	-0.08	1.01	-0.38	-0.29	0.23	0.23	-0.73	-0.28	-2.08	-0.11
JZL610	PH6WC	3.15	3.05	0.08	-0.17	-0.57	0.29	0.23	0.55	0.06	0.14	0.00	-0.67	0.47	0.17	0.26	0.12
Chang7_2	PH6WC	-2.25	2.09	0.02	-0.07	-0.35	2.20	0.16	1.99	0.45	-0.30	-0.92	0.40	-0.22	0.05	-1.16	-0.12
KH786	PH6WC	23.21	4.37	2.88	0.27	3.45	-1.26	0.49	3.50	0.81	-0.02	1.04	0.07	2.38	1.09	2.71	0.19
KH467	PH6WC	-6.43	-2.96	-0.14	-0.04	-0.54	-0.23	-0.35	0.50	0.15	-0.37	0.14	1.19	0.60	-0.14	0.90	-0.12
773_2G	PH6WC	-6.23	-1.47	0.38	-0.02	2.07	1.40	0.15	2.96	0.07	0.39	-0.16	0.58	0.40	0.23	-0.21	0.02
Zha461	PH6WC	-16.46	6.35	0.18	0.02	-0.30	-1.34	0.95	2.33	0.78	0.04	-0.69	-1.81	-1.55	0.15	0.60	-0.11
917	PH6WC	0.66	-3.41	0.09	-0.15	-0.92	0.28	-0.46	2.10	-0.40	0.09	-0.13	2.31	-0.18	0.38	-0.52	-0.05
Hei2	PH6WC	5.56	1.72	-0.33	-0.10	0.67	-0.13	-0.32	0.02	-0.26	-0.17	-0.36	3.25	-0.24	0.04	-0.43	-0.06
K454	PH6WC	2.70	-3.44	0.57	-0.10	-0.39	-0.59	-0.07	-0.11	-0.29	0.15	0.52	1.23	1.23	0.05	-0.36	-0.06
M502	PH6WC	-17.75	-5.74	-1.06	0.02	-3.24	-0.24	0.94	-2.90	0.83	0.14	-0.33	-3.62	0.25	0.23	-0.83	-0.14
7857	PH6WC	-5.92	-5.51	0.18	-0.05	1.23	1.35	0.41	-0.08	-0.34	0.14	0.34	-1.21	0.15	-0.15	-1.87	0.01
Xin444	PH6WC	1.75	3.56	-0.12	-0.06	-0.16	0.62	-0.24	-0.36	0.11	-0.35	0.02	0.54	-0.57	0.57	-0.06	-0.07
Zong31	PH6WC	4.03	2.43	-0.36	-0.08	-0.99	-1.27	-0.37	-0.24	-0.12	0.33	-0.40	-0.57	-0.20	0.54	-0.24	-0.07
X178	PH6WC	-2.88	-1.41	-0.24	0.08	0.63	-0.22	0.15	0.18	0.19	-0.39	0.76	-0.04	0.18	0.83	0.12	-0.04
HuangC	PH6WC	-11.25	-4.79	-0.18	0.00	-0.48	2.71	-0.03	-0.64	0.22	0.26	-2.16	-0.66	1.97	-0.28	1.02	0.01
P138	PH6WC	0.22	3.27	-0.69	0.06	-1.17	0.04	0.04	-0.36	-0.12	-0.19	0.91	0.76	-0.83	0.01	0.75	0.09
Zhong451	PH6WC	-6.90	-0.51	-0.38	0.13	-0.10	1.03	0.62	-1.67	0.08	0.01	0.66	0.78	-0.57	-0.17	0.61	0.04

ZZ01	PH6WC	-1.03	-8.17	-0.09	-0.07	0.82	0.36	-0.05	-0.24	0.12	-0.01	-0.31	-0.05	0.17	-0.17	0.25	-0.13
HZS	PH6WC	3.79	-0.47	-0.09	-0.06	-0.07	2.39	0.04	0.19	0.21	0.14	-0.04	-0.21	0.19	-0.12	1.52	0.02
5213	PH6WC	-1.10	-2.13	0.13	0.03	-1.31	0.25	0.03	-1.12	0.19	-0.08	0.79	-0.47	-0.75	-0.48	1.03	0.05
CN165	PH6WC	-7.31	-0.03	-1.86	0.04	-1.69	0.64	0.47	-0.78	-0.02	-0.20	0.23	-0.18	-0.43	0.28	-1.04	-0.09
CN4379	PH6WC	-11.46	-10.68	-1.17	0.07	-0.31	1.13	0.06	-1.02	0.21	0.10	0.21	-0.85	-0.01	-0.10	0.05	-0.04
Qi318	PH6WC	-3.91	-3.91	-0.46	0.00	0.34	-0.48	0.40	1.37	0.03	0.03	0.06	-0.85	0.56	-0.72	0.28	-0.10
YFH	PH6WC	5.96	1.69	0.34	-0.01	0.64	2.52	0.00	0.59	-0.15	-0.09	0.35	0.84	-0.64	-0.12	-1.80	-0.06
P007	PH6WC	1.39	-2.67	0.27	-0.04	1.64	0.20	-0.21	0.87	0.08	0.06	1.19	0.67	-0.29	-0.19	1.22	0.00
F349	PH6WC	-0.44	-4.49	0.49	-0.03	1.64	0.50	-0.28	-0.29	-0.36	-0.03	0.68	-0.31	-0.19	-0.52	-1.56	0.04
P25	PH6WC	-10.84	-10.17	-1.20	0.04	-0.62	-0.63	-0.40	-0.06	-0.13	-0.28	0.96	-1.09	-0.53	-0.13	0.10	-0.05
Qi205	PH6WC	6.03	1.66	0.60	-0.05	-0.27	0.86	-0.36	0.34	0.00	-0.02	-0.06	0.19	0.38	-0.81	1.07	-0.01
CA375	PH6WC	-2.44	0.05	-0.59	0.01	0.02	1.87	0.19	-0.36	-0.15	0.13	0.54	0.34	0.13	-0.12	0.95	-0.07
Jing501	PH6WC	-7.98	-3.16	-0.45	0.03	-0.61	-0.53	0.05	1.92	-0.23	-0.14	0.72	-0.50	-0.68	0.27	0.64	-0.08
Jing24	PH6WC	8.47	0.89	0.71	-0.01	1.13	0.93	-0.01	-0.67	0.17	-0.41	-0.55	0.82	0.47	-0.36	0.26	-0.02
Jing89	PH6WC	-3.90	-3.74	-0.15	-0.06	0.74	1.31	0.19	0.96	-0.09	-0.35	0.16	-1.27	0.89	-0.08	-1.58	-0.04
Jing572	PH6WC	0.39	-2.64	0.46	-0.01	0.23	0.66	0.03	0.52	-0.13	0.16	0.58	-0.50	-0.10	-0.35	-0.22	-0.03
NH60	PH6WC	-17.71	-6.10	-0.27	-0.08	-1.77	-0.48	-0.04	-0.49	-0.44	-0.09	-0.33	1.61	-1.58	0.06	-0.02	-0.07
Jing724	PH6WC	-15.25	-9.29	-0.40	-0.10	-2.67	-0.51	0.22	-2.50	-0.31	-0.19	-0.59	1.60	-1.59	0.33	-0.22	-0.20
Jing92	PH6WC	0.43	-1.06	0.41	-0.02	0.49	1.70	0.22	1.40	0.17	-0.06	-1.39	0.00	-0.31	-0.38	-1.60	-0.05
Jing725	PH6WC	-11.64	-5.68	-0.16	0.04	-1.54	-1.50	0.51	-0.50	-0.11	-0.22	-1.14	1.53	-0.22	-0.39	-0.71	-0.07
Aijing525	PH6WC	-0.53	1.75	-1.02	0.14	-2.70	0.65	0.33	-0.08	0.31	0.21	1.09	3.92	-0.55	-0.20	1.77	0.01
C103	PH6WC	8.52	3.27	0.27	-0.03	-0.46	2.12	0.13	0.99	-0.17	-0.04	-0.64	-0.77	-0.18	-0.03	-1.38	0.07
Zheng58	PH6WC	0.02	-0.45	-0.61	-0.02	-0.89	-0.13	-0.11	1.40	0.48	0.53	0.04	-0.37	0.63	0.46	2.63	-0.17
XL21	PH6WC	5.15	4.66	-0.63	0.00	0.41	-0.80	0.33	-0.59	0.47	-0.02	-0.03	0.25	-0.53	0.88	1.36	0.09
H2671	PH6WC	-11.36	-4.04	-2.06	-0.17	-0.62	-0.79	-0.53	-0.99	0.15	0.65	-0.92	-1.30	3.30	1.02	-0.98	0.07
CL11	PH6WC	-1.84	-1.53	0.09	-0.06	-0.22	0.25	0.03	0.10	-0.21	0.06	0.09	2.19	0.43	-0.03	0.48	-0.12
NG5	PH6WC	0.09	-1.69	-1.42	0.12	-0.09	-1.54	0.21	0.40	-0.07	-0.40	0.92	-0.97	0.29	-0.79	-0.84	-0.01
HD568	PH6WC	5.29	0.73	0.75	0.05	0.98	-0.63	-0.93	0.72	0.23	0.25	-0.36	0.40	0.29	-0.07	0.57	0.09
11DM124	PH6WC	-15.02	-1.14	-1.30	-0.10	-2.73	-0.82	0.66	1.76	-0.37	-0.26	0.01	-1.20	-1.31	-0.04	-2.66	-0.12
CA616	PH6WC	33.68	8.55	3.82	0.29	5.59	-1.78	-0.15	1.28	0.70	0.30	0.67	-0.80	1.99	0.28	3.41	0.20
Ji6003	PH6WC	-0.72	-1.99	-1.00	0.30	2.89	0.29	1.11	3.17	0.38	0.64	1.15	-2.57	0.61	-1.11	1.26	0.16
Ming2325	PH6WC	1.93	4.04	-0.13	0.14	-2.04	-0.32	0.62	2.13	0.09	-0.23	0.77	-0.15	-0.13	0.87	0.72	-0.14
S5137	PH6WC	1.79	1.71	0.39	0.10	0.72	0.40	-0.17	0.90	0.08	0.03	0.32	-1.20	-0.27	0.50	0.36	0.14
F2001	PH6WC	-15.06	-2.31	-1.00	-0.27	-2.13	0.06	0.00	-2.96	-0.15	0.20	-0.53	0.02	0.88	1.28	0.68	-0.12
20143	PH6WC	-0.39	-0.31	0.03	0.00	-0.34	1.76	0.49	1.65	0.14	-0.17	0.27	0.57	-0.24	0.03	-0.22	-0.12
Cheng60	PH6WC	5.56	13.95	1.45	0.04	0.55	-0.32	0.75	-0.60	0.41	0.41	1.04	2.03	1.25	1.24	5.77	0.43
Cheng53	PH6WC	1.33	2.33	0.26	0.00	-0.29	1.36	-0.60	0.03	0.04	0.13	1.20	0.04	-0.46	0.52	0.96	0.09
Chong72	PH6WC	10.14	4.83	-0.65	-0.02	-0.86	0.95	-0.34	1.30	-0.12	-0.02	-0.38	-0.57	-0.86	0.99	0.44	0.00
NP01200	PH6WC	-2.50	0.93	-0.63	-0.38	-0.94	0.45	0.44	-0.02	0.01	0.29	-0.11	0.03	-0.48	0.63	-0.80	-0.16
7884_Ht	PH4CV	-2.22	1.12	0.02	-0.27	-0.22	-1.43	0.42	1.89	-0.39	-0.09	-0.93	-0.35	0.34	0.17	-1.89	-0.03
H2671	PH4CV	-16.65	2.88	-0.72	-0.04	1.64	1.07	0.23	1.54	0.23	0.26	0.13	-3.86	0.63	-0.98	-2.21	-0.03
7884_Ht	PH6WC	2.26	-0.99	0.16	0.19	0.28	0.04	-0.26	-1.55	0.01	0.06	0.36	1.16	-0.17	0.01	1.51	-0.02

Tab. S3 218 The name of major maize inbred lines.

Line	Line	Line	Line	Line	Line	Line	Line
Dong46	Aijing525	M53	L237	CH382	KL2	7857	Dan598
A801	PH6AT	LK11	ZaC546	PH6JM	KL6	Xin444	XF
Liao68	He344	S127	70_104	Dan6263	D3_1	Zong31	634150
LIao3180	C103	Liao8821	L105	C260	D5_2	X178	TM
K12	Zheng58	Shen391	Zi330	C168	HR30	HuangC	M407
Shen151	XL21	Shen8078	446	Liao7980	KWS10_73	P138	F62
Dan717	H2671	Tie84	Ji853	F12	PHB1M	Zhong451	Dan340
Liao8478	CL11	Lv9W	434	1134	KWS49	ZZ01	DG11A
Zheng22	NG5	9137	4F1	K0325	Jia33	Long53	Si428
Zhong106	HD568	391	444	2511A	Jia28	HZS	Cheng18
Liao3162	11DM124	XKZ101_1	Ji002	CH382	KW5G321	5213	Si144
Dong237	CA616	XKZA34	Ying64	Z3_87	KW1A139	CN165	HYM
PH09B	Ji6003	KL3	S8_101	M60	H1208	CN4379	Lv28
PHPMO	8941	XKZ167_1	7922	S121	L203	Qi318	E28
Dan988	Ming2325	GLMo17	7884_7Ht	P2237	81162	YFH	Mo17Ht
DanT138	S5137	JZL610	96478	K3841	8638	P007	Shen5003
Liao6082	F2001	Chang7_2	Ji992	SD7928	833	F349	KYC8605
63792	20143	KH786	Ji1037	SD8738	ZhongM_8	P25	7_61
7017	Cheng60	KH467	Si_287	K10	9F592	Qi205	Fu746Mu
Dan99C	Cheng53	773_2G	W9706	Shen3117	6F576	Fu706	Liao2345
Shen3336	Chong72	Zha461	8902	Dan1133	KL613	CA375	Q1261
Shen3265	NP01200	917	Cheng351	Dan37	385_1	Jing501	Shen137
Hai268	7884-Ht	Hei2	Dan891	M03	HA25	Jing24	Dan9046
T98103_1	Dong6002	KL4	D22	M5972	KL45	Jing89	8112
Dan360	H2671	K454	KX	Ming84	Ji63	Jing572	Liao1412
CL143	Ji846	M502	Ji046	Ming71	Men14	NH60	LD175_1
T0278	Xi14	Jing724	LD61	Jing725	Dan3130	Jing92	D26
TieT0403	Mo17						

Tab. S4 436 The name of hybrid combinations.

Cross	Cross	Cross	Cross	Cross	Cross	Cross	Cross
XF×PH6WC	PH6AT×PH6WC	Liao3162×PH4CV	PHPMO×PH6WC	Ji853×PH4CV	434×PH6WC	Chang7_2×PH4CV	KH467×PH6WC
PH6AT×PH4CV	PHB1M×PH6WC	PH09B×PH4CV	Dan988×PH6WC	434×PH4CV	4F1×PH6WC	KH786×PH4CV	773_2G×PH6WC
PHB1M×PH4CV	ZhongM_8×PH6WC	PHPMO×PH4CV	DanT138×PH6WC	4F1×PH4CV	444×PH6WC	KH467×PH4CV	Zha461×PH6WC
ZhongM_8×PH4CV	L237×PH6WC	Dan988×PH4CV	Liao6082×PH6WC	444×PH4CV	Ji002×PH6WC	773_2G×PH4CV	917×PH6WC
L237×PH4CV	Ying64×PH6WC	DanT138×PH4CV	63792×PH6WC	Ji002×PH4CV	S8_101×PH6WC	Zha461×PH4CV	Hei2×PH6WC
Ying64×PH4CV	Dan891×PH6WC	Liao6082×PH4CV	7017×PH6WC	S8_101×PH4CV	7922×PH6WC	917×PH4CV	K454×PH6WC
Dan891×PH4CV	DG11A×PH6WC	63792×PH4CV	Dan99C×PH6WC	7922×PH4CV	7884_7Ht×PH6WC	Hei2×PH4CV	M502×PH6WC
DG11A×PH4CV	Fu746Mu×PH6WC	7017×PH4CV	Shen3336×PH6WC	7884_7Ht×PH4CV	96478×PH6WC	K454×PH4CV	7857×PH6WC
Fu746Mu×PH4CV	Dong46×PH6WC	Dan99C×PH4CV	Shen3265×PH6WC	96478×PH4CV	Ji992×PH6WC	M502×PH4CV	Xin444×PH6WC
Dong46×PH4CV	Dong237×PH6WC	Shen3336×PH4CV	T98103_1×PH6WC	Ji992×PH4CV	Ji1037×PH6WC	7857×PH4CV	Zong31×PH6WC

Dong237×PH4CV	Hai268×PH6WC	Shen3265×PH4CV	Dan360×PH6WC	Ji1037×PH4CV	Si_287×PH6WC	Xin444×PH4CV	X178×PH6WC
Hai268×PH4CV	1134×PH6WC	T98103_1×PH4CV	CL143×PH6WC	Si_287×PH4CV	W9706×PH6WC	Zong31×PH4CV	HuangC×PH6WC
1134×PH4CV	K10×PH6WC	Dan360×PH4CV	CH382×PH6WC	W9706×PH4CV	8902×PH6WC	X178×PH4CV	P138×PH6WC
K10×PH4CV	LK11×PH6WC	CL143×PH4CV	PH6JM×PH6WC	8902×PH4CV	Cheng351×PH6WC	HuangC×PH4CV	Zhong451×PH6WC
LK11×PH4CV	KL3×PH6WC	CH382×PH4CV	Dan6263×PH6WC	Cheng351×PH4CV	D22×PH6WC	P138×PH4CV	ZZ01×PH6WC
KL3×PH4CV	KL4×PH6WC	PH6JM×PH4CV	C260×PH6WC	D22×PH4CV	KX×PH6WC	Zhong451×PH4CV	HZS×PH6WC
KL4×PH4CV	Long53×PH6WC	Dan6263×PH4CV	C168×PH6WC	KX×PH4CV	Ji046×PH6WC	ZZ01×PH4CV	5213×PH6WC
Long53×PH4CV	Fu706×PH6WC	C260×PH4CV	Liao7980×PH6WC	Ji046×PH4CV	Dan598×PH6WC	HZS×PH4CV	CN165×PH6WC
Fu706×PH4CV	He344×PH6WC	C168×PH4CV	F12×PH6WC	Dan598×PH4CV	634150×PH6WC	5213×PH4CV	CN4379×PH6WC
He344×PH4CV	8941×PH6WC	Liao7980×PH4CV	K0325×PH6WC	XF×PH4CV	TM×PH6WC	CN165×PH4CV	Qi318×PH6WC
8941×PH4CV	Dong6002×PH6WC	F12×PH4CV	2511A×PH6WC	634150×PH4CV	M407×PH6WC	CN4379×PH4CV	YFH×PH6WC
Dong6002×PH4CV	Ji846×PH6WC	K0325×PH4CV	CH382×PH6WC	TM×PH4CV	F62×PH6WC	Qi318×PH4CV	P007×PH6WC
Ji846×PH4CV	KL2×PH6WC	2511A×PH4CV	Z3_87×PH6WC	M407×PH4CV	Dan340×PH6WC	YFH×PH4CV	F349×PH6WC
KL2×PH4CV	KL6×PH6WC	CH382×PH4CV	M60×PH6WC	F62×PH4CV	Si428×PH6WC	P007×PH4CV	P25×PH6WC
KL6×PH4CV	D3_1×PH6WC	Z3_87×PH4CV	S121×PH6WC	Dan340×PH4CV	Cheng18×PH6WC	F349×PH4CV	Qi205×PH6WC
D3_1×PH4CV	D5_2×PH6WC	M60×PH4CV	P2237×PH6WC	Si428×PH4CV	Si144×PH6WC	P25×PH4CV	CA375×PH6WC
D5_2×PH4CV	HR30×PH6WC	S121×PH4CV	K3841×PH6WC	Cheng18×PH4CV	HYM×PH6WC	Qi205×PH4CV	Jing501×PH6WC
HR30×PH4CV	KWS10_73×PH6WC	P2237×PH4CV	SD7928×PH6WC	Si144×PH4CV	Lv28×PH6WC	CA375×PH4CV	Jing24×PH6WC
KWS10_73×PH4CV	KWS49×PH6WC	K3841×PH4CV	SD8738×PH6WC	HYM×PH4CV	E28×PH6WC	Jing501×PH4CV	Jing89×PH6WC
KWS49×PH4CV	Jia33×PH6WC	SD7928×PH4CV	Shen3117×PH6WC	Lv28×PH4CV	Mo17Ht×PH6WC	Jing24×PH4CV	Jing572×PH6WC
Jia33×PH4CV	Jia28×PH6WC	SD8738×PH4CV	Dan1133×PH6WC	E28×PH4CV	Shen5003×PH6WC	Jing89×PH4CV	NH60×PH6WC
Jia28×PH4CV	KW5G321×PH6WC	Shen3117×PH4CV	Dan37×PH6WC	Mo17Ht×PH4CV	KYC8605×PH6WC	Jing572×PH4CV	Jing724×PH6WC
KW5G321×PH4CV	KW1A139×PH6WC	Dan1133×PH4CV	M03×PH6WC	Shen5003×PH4CV	7_61×PH6WC	NH60×PH4CV	Jing92×PH6WC
KW1A139×PH4CV	H1208×PH6WC	Dan37×PH4CV	M5972×PH6WC	KYC8605×PH4CV	Liao2345×PH6WC	Jing724×PH4CV	Jing725×PH6WC
H1208×PH4CV	L203×PH6WC	M03×PH4CV	Ming84×PH6WC	7_61×PH4CV	Q1261×PH6WC	Jing92×PH4CV	Aijing525×PH6WC
L203×PH4CV	81162×PH6WC	M5972×PH4CV	Ming71×PH6WC	Liao2345×PH4CV	Shen137×PH6WC	Jing725×PH4CV	C103×PH6WC
81162×PH4CV	8638×PH6WC	Ming84×PH4CV	T0278×PH6WC	Q1261×PH4CV	Dan9046×PH6WC	Aijing525×PH4CV	Zheng58×PH6WC
8638×PH4CV	833×PH6WC	Ming71×PH4CV	TieT0403×PH6WC	Shen137×PH4CV	8112×PH6WC	C103×PH4CV	XL21×PH6WC
833×PH4CV	9F592×PH6WC	T0278×PH4CV	M53×PH6WC	Dan9046×PH4CV	Liao1412×PH6WC	Zheng58×PH4CV	H2671×PH6WC
9F592×PH4CV	6F576×PH6WC	TieT0403×PH4CV	S127×PH6WC	8112×PH4CV	LD175_1×PH6WC	XL21×PH4CV	CL11×PH6WC
6F576×PH4CV	KL613×PH6WC	M53×PH4CV	Liao8821×PH6WC	Liao1412×PH4CV	LD61×PH6WC	H2671×PH4CV	NG5×PH6WC
KL613×PH4CV	385_1×PH6WC	S127×PH4CV	Shen391×PH6WC	LD175_1×PH4CV	D26×PH6WC	H2671×PH4CV	HD568×PH6WC
385_1×PH4CV	HA25×PH6WC	Liao8821×PH4CV	Shen8078×PH6WC	LD61×PH4CV	Dan3130×PH6WC	CL11×PH4CV	11DM124×PH6WC
HA25×PH4CV	KL45×PH6WC	Shen391×PH4CV	Tie84×PH6WC	D26×PH4CV	A801×PH6WC	NG5×PH4CV	CA616×PH6WC
KL45×PH4CV	Ji63×PH6WC	Shen8078×PH4CV	Lv9W×PH6WC	Dan3130×PH4CV	Liao68×PH6WC	HD568×PH4CV	Ji6003×PH6WC
Ji63×PH4CV	Men14×PH6WC	Tie84×PH4CV	9137×PH6WC	A801×PH4CV	LIao3180×PH6WC	11DM124×PH4CV	Ming2325×PH6WC
Men14×PH4CV	Xi14×PH6WC	Lv9W×PH4CV	391×PH6WC	Liao68×PH4CV	K12×PH6WC	CA616×PH4CV	S5137×PH6WC
Xi14×PH4CV	Mo17×PH6WC	9137×PH4CV	XKZ101_1×PH6WC	LIao3180×PH4CV	Shen151×PH6WC	Ji6003×PH4CV	F2001×PH6WC
Mo17×PH4CV	ZaC546×PH6WC	391×PH4CV	XKZA34×PH6WC	K12×PH4CV	Dan717×PH6WC	Ming2325×PH4CV	20143×PH6WC
ZaC546×PH4CV	70_104×PH6WC	XKZ101_1×PH4CV	XKZ167_1×PH6WC	Shen151×PH4CV	Liao8478×PH6WC	S5137×PH4CV	Cheng60×PH6WC
70_104×PH4CV	L105×PH6WC	XKZA34×PH4CV	GLMo17×PH6WC	Dan717×PH4CV	Zheng22×PH6WC	F2001×PH4CV	Cheng53×PH6WC
L105×PH4CV	Zi330×PH6WC	XKZ167_1×PH4CV	JZL610×PH6WC	Liao8478×PH4CV	Zhong106×PH6WC	20143×PH4CV	Chong72×PH6WC

Zi330×PH4CV	446×PH6WC	GLMo17×PH4CV	Chang7_2×PH6WC	Zheng22×PH4CV	Liao3162×PH6WC	Cheng60×PH4CV	NP01200×PH6WC
446×PH4CV	Ji853×PH6WC	JZL610×PH4CV	KH786×PH6WC	Zhong106×PH4CV	PH09B×PH6WC	Cheng53×PH4CV	7884_Ht×PH4CV
NP01200×PH4CV	7884_Ht×PH6WC	Chong72×PH4CV	H2671×PH4CV				
